# A qualitative study investigating Stakeholders' perspectives on a professional body of pharmacy

**DOI:** 10.1016/j.rcsop.2022.100170

**Published:** 2022-08-19

**Authors:** Peter O'Sullivan, Suzanne McCarthy

**Affiliations:** a School of Pharmacy, University College Cork, Cork, Ireland

**Keywords:** Professionalism, Pharmacy, Representation, Regulation, Qualitative research

## Abstract

**Background:**

With the advent of “shared regulation” over a decade ago in healthcare to allow for greater public input, the Pharmaceutical Society of Ireland (PSI) shed its professional leadership role. Since then there has been no unified voice for the profession of pharmacy in Ireland, which is in stark contrast to other jurisdictions and allied healthcare professions, where both public and practitioner are catered for in separate entities. This is an issue which has received little academic scrutiny thus far, and therefore this study provides a unique opportunity for stakeholders to submit their views.

**Methods:**

Semi-structured interviews with key stakeholders working in representative bodies in Ireland using purposive and snowball sampling. Each interview was audio-video recorded and transcribed accordingly for six phase thematic analysis.

**Results:**

Interviews were conducted with thirteen participants working in diverse sectors relevant to professional representation. There was a consensus regarding the existing void in the pharmacy profession, and how this has had a detrimental impact on the development of the profession and pharmacists' position in the Irish healthcare system. Different structural models were proposed by participants and potential financial and logistical hurdles for the profession to overcome were considered.

**Conclusion:**

The study provides a unique review of stakeholders' perspectives who had clear desires for change. The manner in which such change will occur is a consideration for the profession and policy makers going forward.

## 1 Introduction

The last two decades saw a seismic shift in the pharmacy profession, following the lead of other healthcare professions such as medicine and nursing. It underwent a transformation from *“self-regulation”,* where the profession itself sets regulatory standards, to a *“shared”* regulatory approach, where public input is a mandatory requirement for any regulation.^1^ It was seen that a clear demarcation between the profession and the public was necessary following several inquiries into a string of medical malpractice scandals in the healthcare sector in the late 1990s in the UK, e.g. Kennedy and Shipman Inquiry.^2^ These inquiries criticised the sense of collegiality and *“club-like”* mentality on the part of healthcare professionals to the detriment of the public interest.^2^ The new era regulators that emerged were independent bodies devoid of any professional obligations, with sole allegiance to the public mandate.^3^ The White Paper 2007 was the catalyst for change in the UK,^4^^,^^5^ where the Royal Pharmaceutical Society of Great Britain (RPSGB) with its dual *regulatory* and *professional leadership* role was separated into the General Pharmaceutical Council (GPhC)(*independent regulator)*,^6^ and Royal Pharmaceutical Society (RPS)(*professional representative*).^7^ One of the hallmarks of addressing the issues with *“self-regulation”* is encompassing an equal number of lay-members to the GPhC Council design.^8^ The RPS's domain on the other hand was to *lead, recognize, support, develop, network* and *inform* the profession in an effort to provide a pillar to the profession with their new regulator.^9^

The pharmacy sector in Ireland influenced by the UK philosophy and endogenous instances of medical misconduct, e.g. Lourdes Hospital Inquiry,^10^ evolved in a manner similar to their UK counterparts in the guise of the Pharmacy Act 2007.^11^ Prior to the enactment of this Act, there was little scope for the removal of an aberrant pharmacist from the register. This was due to the *“widespread”* acceptance of the non-regulatory professional leadership and development role of the old-Pharmaceutical Society of Ireland (PSI), and the constitutionally protected right of a registrant to earn a livelihood.^12^ The new suite of disciplinary provisions in the 2007 Act were again something unfamiliar to members of the previous incarnation of the PSI.^13^ The PSI, divested of its professional prerogative, is now the sole regulator for the profession with a majority lay Council (11:10), but unlike the UK, there was no simultaneous establishment of a complementary body exclusive to the profession.^14^ This has been seen as a serious failing which has *“weakened the profession's advocacy”* abilities for inclusion in the healthcare system and its policy initiatives^15^ and also potentially in the eyes of the public.^16^ Table 1 outlines the various bodies in existence in Ireland at present. There are currently partial representative bodies in Ireland, through trade unions, namely the Irish Pharmacy Union (IPU),^17^ and the Hospital Pharmacist Association of Ireland (HPAI).^18^ Both of these are sector specific and stymied by certain contractual obligations of their trade union status, and therefore cannot be considered an all-encompassing *“voice”* for the profession.^14^^,^^15^ The regulator has made attempts to enhance its professional leadership remit, namely through educational endeavours in appointing the Irish Institute of Pharmacy(IIOP) as the *“managing body*” for Continuous Professional Development (CPD) in 2013.^19^^,^^20^ However, this was systematically and independently reviewed five year ago and confusion remains as to whether it is a *quasi* “*professional body”* or merely a de facto emanation of the regulator due to its limited funding and autonomy to solely service CPD on behalf of the PSI.^21^

**Table 1:** Current key professional bodies in Ireland.

Name	Founded	Purpose	Membership	Membership Type
Irish Pharmacy Union (IPU)^17^	1973	Representative	Registered and dispensing chemists and registered druggists, who are registered as community pharmacists with the Pharmaceutical Society of Ireland	Voluntary – Paid membership
Hospital Pharmacists Association of Ireland (HPAI)^18^	1977	Representative	Hospital pharmacists working in Ireland.	Voluntary – Paid membership
Pharmaceutical Society of Ireland (PSI)^11^	2007	Regulatory	Pharmacists and pharmaceutical assistants must be registered with PSI in order to practice in Ireland.	Mandatory – Paid membership
Pharmacists in Industry and Education and Regulation (PIER)^27^	2012	Representative	Open to anyone who has undertaken a pharmacy degree (in Ireland or other countries) and is eligible to be listed on the Register of the PSI.	Voluntary – Paid membership
Irish Institute of Pharmacy (IIOP)^19^^,^^20^	2013	Educational	All pharmacists listed on the Register of the PSI.	Mandatory – No payment for pharmacists
Affiliation for Pharmacy Practice Experiential Learning (APPEL)^27^	2015	Educational	Oversees and supports experiential learning placements for all students in the three Schools of Pharmacy across Ireland.	Mandatory – No payment for students

It has now been 15 years since the Pharmacy Act's enactment, and the issue of an appropriate professional agenda to unite all pharmacists is something which still lies unresolved and continues to vex the profession to this day. Some consider the legislation to signal the creation of a “deterrence” regulator,^22^ and others fear the potential fostering of defensive and closed practices among pharmacists in an effort to avoid litigation.^23^ With this in mind, it is lamentable how little academic writing has since been carried out on this existing professional vacuum in Ireland for pharmacy.^14^ This study is an effort to address this need and aims to understand the perceptions of individual key stakeholders on the current representative landscape for pharmacy, and if there is sufficient appetite for a professional entity to potentially be implemented and integrated in Ireland to ameliorate this.

## 2 Methods

### 2.1 Study design and ethical approval

A qualitative description (QD) study was undertaken; QD aims to present a description and comprehensive summary of the phenomenon of interest, in this case a professional body for pharmacy in Ireland, using participants' language and interpretation that is low-inference.^24^ Data were collected using semi-structured interviews as this provided for more open-ended discussion on the topic,^25^ and was not subject to the same “biases” or *“groupthink”* mentality that can occur when researchers attempt to moderate focus groups.^26^ Participant inclusion was sought from individuals who had experience working currently in representative bodies in Ireland, namely the IIOP, Appel, IPU, HPAI, PIER^27^ and the three Schools of Pharmacy in Ireland (University College Cork, Trinity College Dublin and Royal College of Surgeons in Ireland). Suitable individuals were invited by email to participate with details of the study (i.e. information sheet) and a consent form provided. Ethical approval was obtained from the Social Research Ethics Committee of University College Cork.^28^ The concept of data saturation, as operationalised by Francis et al. was used when considering sample size for this study.^29^ It was planned in advance that if no new themes were identified in the additional 3 interviews (stopping criterion) after the 10th (initial analysis sample), then this would confirm that the topic had been thoroughly explored. The stopping criterion was tested after each successive interview, e.g. 11, 12, 13; the authors were satisfied that no new themes were generated during these interviews and thus further interviews were not required.^29^ The participants were identified initially using a combination of *purposive* sampling from the aforementioned bodies' websites, and follow-on contacts provided from initial interviewees (i.e. *snowball* sampling).^30^

All aspects in reporting this study were guided by the 32 item check list of the Consolidated Criteria for Reporting Qualitative Research (COREQ) statement.^31^ (see Appendix B).

### 2.2 Data collection

An “interview topic guide” was drafted by the authors (POS and SM) based on existing literature on the topic^14^^,^^15^ and their own perception of salient issues needing scrutiny. The face and content validity of the guide was examined by the authors through conducting a pilot interview with SM as the interviewee, and areas for improvement in the format were ascertained prior to the commencement of the participant recruitment phase. During the research process, the guide was revised slightly to allow interesting points of inquiry, revealed from interviewees, to be incorporated in subsequent interviews. For example, the question of leadership qualities for a professional entity was included following the initial interviews. (see guide in Appendix A). This helped maintain the spirit of the semi-structured nature of the research.^25^

Each participant was contacted by email in advance of the interview to provide them with adequate time to process the information sheet, and return the completed consent form. In order to ensure the anonymity of all participants, demographics were noted prior to recording (*infra* Table 2, Results). All interviews were conducted using Microsoft® Teams. Participants and interviewer cameras were used during the interviews to provide greater engagement. The audio and video data were recorded on Microsoft Teams; the visual data were not used for analysis purposes. Each interview was then transcribed *verbatim* with the assistance of Microsoft Teams inbuilt transcription software, with subsequent careful revision and cross-checking of each transcript by both authors. Interviewees were afforded the opportunity to review said transcripts, and withdraw their consent within 14 days, if they so wished.

**Table 2:** Participant demographics.

Characteristic	No (*n* = 13)
Gender	
Female	7
Male	6
Years post registration	
>20 years	11
>15 years	1
>10 years	1
Practice	
Patient facing	4
Non-patient facing	9
Roles⁎	
Professional representation and development	6
Education	5
Practice in community and hospital	4
List of organizations affiliated to participants	Appel; Health Service Executive; HPAI; Hospital Pharmacy; IIOP; IPU; Trinity College Dublin; University College Cork

⁎ Two participants had multiple roles and therefore n = 13 does not apply in this instance.

### 2.3 Data analysis

**Fig. 1:**
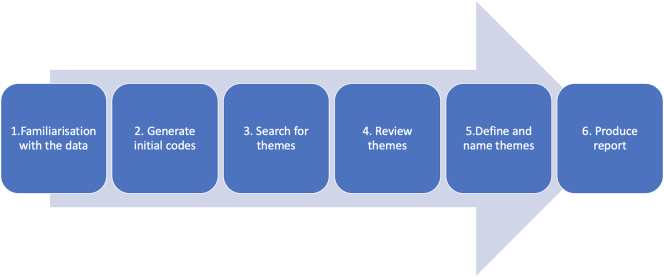
Clarke and Braun 6 Phases of Thematic Analysis.^32^

After the interviews were transcribed, thematic analysis in line with the six phase method outlined by Clarke and Braun was carried out.^32^ The details of this thematic analysis are provided in Fig. 1. The transcripts were re-read several times by the author to ensure *familiarity* with the data (Phase 1). Following this, inductive *coding* was carried out without prior categorisation so that themes could be generated after analysis and continuous reflection of the data (*Phase 2*). It was also necessary to include a miscellaneous code so that outlier data could be reviewed later.^33^ These codes were then assembled by the author and potential *themes* were *sought* and reviewed with SM (*Phase 3 and 4*). Following this the *themes* and *subthemes* were finalised (*Phase 5*), and representative quotations were ascribed to each theme in the results section of this report. (*Phase 6*).^32^

The qualitative software used for this process was NVivo® version 1.6.1 (2022).

## 3 Results

The variety of participant demographics are shown in Table 2 below. All interviews took place between the months of November 2021 and January 2022. The length of each interview ranged from 17 to 80 min in duration, with a median value of 31 min.

Four main themes and nine subthemes were identified from the data and are discussed in this section (see Table 3 below).

**Table 3:** Summary of themes and subthemes.

Theme	Subtheme
1*. Reflections on current professional needs* At present there is no all-encompassing representative entity for pharmacy in Ireland. The ramifications of this are explored.	Voids in Representative landscape
	Public Benefit
	Underutilisation of Pharmacist skill-sets
2*. Regulation* Since 2007 the PSI (regulator), which previously provided leadership functions for the profession is now exclusively a regulator.	Independence from the regulator
	Educational Accreditation
3*. Structures and Functions* Four suggested models for fulfilling the professional agenda are discussed (*infra*Table 4). Respective functions and policies are also elaborated, e.g. advocacy & education.	Structures
	Functions & Policies
4. *Finance & Membership* The funding of any professional entity are discussed in detail (*infra*Table 5), and potential membership requirements.	Finance
	Membership & Inclusiveness

For each theme, the relevant subthemes are elaborated upon with pertinent quotations from participants included to exemplify perspectives more clearly. In Appendix C of this review, additional full quotations can be found, which provide greater evidence of interviewees' views on each associated theme and sub-theme.

### 3.1 Theme 1 reflections on current professional needs

#### 3.1.1 Voids in the representative landscape

There was a clear recognition on behalf of participants that there is a need for greater professional representation in pharmacy than the existing “*ad hoc representative”* bodies, which may be perceived as being either too specialized or too trade union oriented:

“*I don't think it's[the current representation] sufficient…..and I think it's very local, and it's very open to individuals as opposed to a profession.” **(Participant 5).***

It was in fact pointed out by some that the roles of the current bodies (*supra* Table 1) could be augmented if this professional need was more catered for:

“*I think they[IPU and HPAI] have done their best to develop it….everybody is doing great work to make huge contributions, but I think it's very much almost on the side of what it could be. So if we had a professional body…they could… carry a lot more traction in their work.” **(Participant 11).***

In terms of pausing to reflect on the past timeline, many participants were critical of the missed opportunity they saw in this professional void not being addressed when the old-PSI rebranded itself as their regulator. While two other participants called for reflection on the more recent professional endeavours of the PSI via the IIOP:

*“we thought that [IIOP] would provide a leadership role for the profession. And I think we need to seriously reflect and see why that hasn't been achieved and realized. Not to make the same mistakes again.” **(Participant 3).***

#### 3.1.2 Public benefit and perception

While not always at the forefront of the discussion in setting a professional agenda, several participants were aware that it is inextricably linked to and provides a consequential societal benefit to patients. In this regard, one participant called for greater clarification of their skill-sets:

*“Pharmacists do an awful lot…that's not seen… we've evolved out of dispensaries, and I think we're so much more than that now….. it's really important that we…start….to show our transparency and what we do as a profession for the public first and foremost, and also for the profession” **(Participant 11).***

A greater public perception of the profession in itself would lead to enhanced trust and awareness among members of the public regarding the various benefits that pharmacists can offer patients. However, in order for this “*societal benefit*” to develop, it is argued that a unified representative body would be required to objectively present this perspective to the public:

*“In setting itself up as a profession it commits to using that knowledge for societal benefit rather than self-interest. So the importance of having professional representation is bringing to bear a collective responsibility for society to use the knowledge across the profession for the benefit of patients in this case. And the lack of professional representation means that what you're left with is representation on different agendas.” **(Participant 1).***

#### 3.1.3 Underutilisation of pharmacist skill-sets

As well as the uncaptured existing elements of the career (e.g. patient counselling, medication reconciliation and seasonal vaccinations) the underutilisation and expansion of the pharmacist's role was a bone of contention and seen by some participants as a ramification of the existing gap in their professional development. Participants gave specific examples where they felt the pharmacist's skills could be used to their *“fullest potentials”,* e.g. GP-practice pharmacists, prescribing pharmacists and also at the transitions of care:

“*Knowing that the WHO patient safety agenda at the moment is looking at polypharmacy transitions of care and high risk situations and there's no pharmacist. It's not because nobody understands the role of pharmacists, it is because there's an ignorance: one there's no pharmacist in there to advocate or to inform, and there's a perception that advanced nurse practitioners can do this work.” **(Participant 1).***

This can be seen as a role that a new professional entity could advocate and lobby to improve upon going forward (*infra Theme 3 Structure and Functions*).

### 3.2 Theme 2 regulation

#### 3.2.1 Independence from regulation

The vast majority of participants considered separation from the regulator to be an essential core construct which safeguards the protection of the public mandate of the PSI, and an impartial voice for the profession of pharmacy to be attainable:

*“Separate, I think it's very important to have, it is almost like church and state, I just think they have to be completely separate, otherwise there are obvious conflicts.” **(Participant 5).***

The difficulties and complexities that can arise from a lack of separation were exemplified by some participants when discussing the IIOP's “*arm's-length”* arrangement. In this, the revenue stream and remit set by the PSI in line with their contract for service was considered *“unhealthy”.*

It was accepted that this separation does not remove the need for both regulator and professional entity to communicate effectively with each other and have a working relationship in the pharmacy sector:

*“I think they should be independent, but I mean they have to be able to talk. You know, there might be someone on PSI board and someone from the PSI on this board, because I think we have to stop treating the PSI as the devil incarnate, and realize you know they have a role to play and that role is to ensure we are the best pharmacists that we can possibly be and …to… protect us…..indirectly from ourselves.” **(Participant 9).***

One participant did consider the separation to be feasible with “*both housed in the same building [PSI]”,* if we were able to *“trust ourselves”* to manage conflicts should they occur (*infra Theme 3 Structure and Functions)*.

It was also observed that professional independence provides reciprocity to the regulator, in that it prevents them overstepping and undertaking roles which would more appropriately be redesignated to a professional body, e.g. drafting guidelines and making representations to Government. The reason these instances occur was described by one participant as an *“Irish solution to an Irish problem”* and is a product of the current legislative drafting [Pharmacy Act 2007], where the regulator was entrusted with a role *“to advance the profession”* [s7 (2)(a)(viii)] as a means to temper the lack of professional leadership:

“*We have the unusual circumstance in which the regulator still has a role in developing the profession. In truth, it really shouldn't. However, we're stuck with it, so that's one of the reasons why the PSI does some of the things that it does.” **(Participant 10).***

#### 3.2.2 Accreditation of continuous professional development

There was uncertainty as to where the appropriate responsibility of CPD as a role would reside. While some had no issue about outsourcing the process entirely to a separate professional or educational institution, others considered it more “*mandatory”* and punitive in nature and therefore ought to remain within the confines of the PSI.

*“[I]f the regulator can entrust some aspects of accreditation to a professional body…that represents a very good ecosystem within the profession, provided that the appropriate assurances and checks and measures are in place.” **(Participant 1).***

*“No, a professional body shouldn't be doing that. It should be providing a framework, it should be providing guidelines, it should be doing that. But the actual accreditation and checking of this should be as I see it, a role of the regulator.” **(Participant 2).***

### 3.3 Theme 3 structure and functions

#### 3.3.1 Structure

There were essentially four options suggested by interviewees: (1)to create a new body; (2)to use an existing organization as a template (namely the IIOP); (3) to form a collective umbrella organization with all existing bodies; and (4)to broaden the scope of the PSI to encompass an advocacy role. Each of these options are summarised in Table 4 below. The illustrative quotations provided are either in favour of (in white) or opposed to (in grey) a particular model.

**Table 4:**
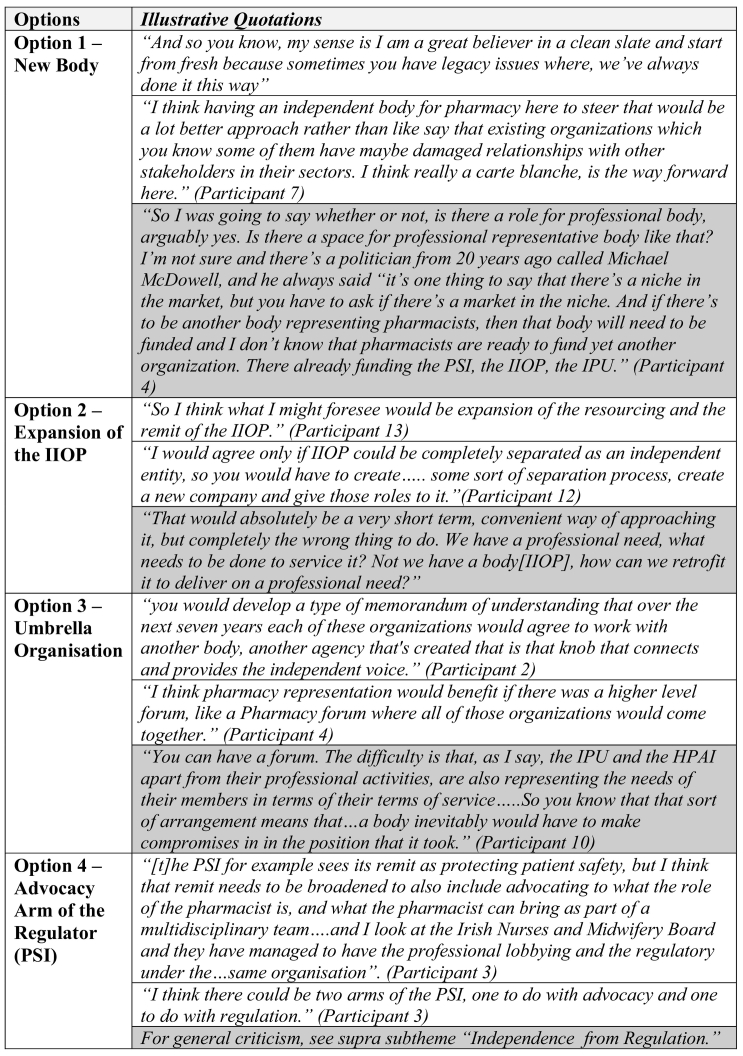
Proposed structures for a professional representative entity with illustrative quotations*.*

There were of course one or two participants who did not express any particular affinity for one model over another, provided the correct inputs and planning was carried out, e.g. making sure not to over-represent from any particular area (e.g. community pharmacists) and an overwhelming preference to have a pharmacist as the leader of such an organization. In terms of who had the correct authority to begin this process, two participants considered it a role for the Department of Health (DOH) or PSI, while several others considered it paramount that the profession itself undertake this duty to lend credibility to any initiative:

*“We don't need anyone's permission to start a professional leadership body that unites the whole gamut of Pharmacy. We only need to self-assemble.” **(Participant 12).***

Other jurisdictions mentioned in which to draw parallels included the UK, Canada, New Zealand and Australia. A couple of participants were steadfast in pointing out the inherent “*differences”* which they felt separated the UK and Ireland, in that the RPS was financially and politically more appealing during its inception due to the assets accrued from RPSGB. In fact, one interviewee considered the RPS to be an *“ivory towers”* entity and overtly *“academic”* to reflect the reality of day-to-day practice. Related medical and nursing approaches were also pondered.

#### 3.3.2 Functions and policies

The roles that were attributed to any of the proposed structure were both abundant and practical and extended from advocacy, leadership, support and educational ambitions.

• It was felt that such a body could “*lobby”* for greater career structure (especially in hospital pharmacy [***Participant 1, 7 & 11***]) and development (e.g. *“horizon scanning”* for new roles[***Participant 5***] (*supra theme 1, subtheme “Underutilisation of Pharmacist skill-sets”*), and payments for cognitive services in community (Medicines Use Review)[***Participant 12***].

• It could *lead* the profession and engage with key stakeholders at a policy level and *lobby* for the reinstatement of a chief pharmacist in the DOH [***Participant 3 & Participant 8***].

• In terms of a *support* function, coaching, self-help and provision of a benevolent fund to members [***Participant 13***].
• Aside from the provision of CPD and its portfolio[***Participant 11***](*supra theme 2, subtheme “Accreditation of CPD”*), other *educational* roles could include the publishing of clinical standards and guidelines [***Participant 2, 6 & 11***], credentialing of specialization in practice [***Participant 11 & 13***], pilot studies and publishing of research findings in a member's journal similar to the old PSI's *Irish Pharmaceutical Journal* [***Participant 12***].

• The awarding of *fellowship* was also a common function which would fall within the ambit of a professional body to give distinction to members who make a significant contribution to the pharmacy profession [***Participant 1, 3, 12 & 13***].

It was emphasised by many that in order to accomplish productively these aims there would need to be strong strategic links and *“recognition”* between the professional organization and the DOH/Health Service Executive (HSE; the body who delivers health services in Ireland) at its foundations:

“*It[Pharmacy] is an unknown unknown to the HSE. Individual pockets exist where.. they value pharmacy….. But then you have the places who….actually don't know what we do. They think we dispense tablets, they're in an office, so why would I get one of those people in the door here.” **(Participant 8).***

### 3.4 Theme 4 finance & membership

#### 3.4.1 Source of funding

Financial viability was deemed by many to be one of initial barriers facing any professional organization ***(Participant 4).*** Many participants believed that to ensure integrity and acceptability of objectives, either all or least a significant proportion of funding should come exclusively from the profession itself. Others perceived this may pose an initial stumbling block for *“buy-in”* and therefore alternative or more mandatory methods may be necessitated (see Table 5*)*.

**Table 5:** Proposed funding sources for representative entity.

• Membership Fees • Statutory Funding, e.g. DOH and HSE • Retail and Hospital Pharmacy Sector • No Fee Required in Umbrella Model (*supra* Option 3 in Table 4) • Postgraduate Training • Alteration of the €380 PSI Fee • IIOP Current Allocation: €600 K (DOH) and €500 K (PSI) • Annual Capitation Tax on the Regulator (PSI), e.g. 20% of membership fee

Different combinations of the above were considered, and while some were apprehensive about the potential for conflict from certain financial providers (namely HSE and PSI), others considered this avoidable with rationale planning akin to the Irish College of Anaesthesiologists:

*“[T]hey have that connection into the HSE where there's a direct kind of a quid pro quo, you know? So the HSE are paying them money, but they can see what they're getting in return[postgraduate training].” **(Participant 11).***

#### 3.4.2 Membership and inclusiveness

While some participants considered membership of this body to be mandatory and included with their PSI registration fee, the predominant view emerging was for optional membership with perhaps some consideration:

“*I don't think mandatory supports membership and buy in. I think it does the opposite….. you will not get engagement you know” **(Participant 13).***

Different membership structures such as tiered gradation was seen as a detail to be unravelled *“down the line”* in the organization's genesis (***Participant 2 and 13***). Participants also alluded to reflecting upon the current leakage of membership experienced by the RPS, so that a new Irish initiative would not fall prey to similar shortcomings (***Participant 4, 5, 10 & 12).***

It was seen by all that the fundamental ethos of membership should be to embrace “*inclusiveness”*:

*“I think the goal and mission would have to be very clear that it's all inclusive, and that one voice is just the same as any other voice.” **(Participant 5).***

*“[Professional Body] that's for anyone who did a degree in pharmacy, basically.” **(Participant 9).***

## 4 Discussion

This study is the first extensive discussion and review of stakeholder perspectives exclusively on the topic of professional representation in the Irish pharmacy sector. While issues of professional development and leadership are often alluded to in PSI consultation processes, these are not typically the primary objective of such policy documents, e.g. *Future Pharmacy Practice in Ireland Meeting Patients' Needs*.^34^ Now the concept of having a professional body to represent the whole of the profession is not something unique and is occurring in many other countries, such as the UK's RPS as outlined above.^9^ This would explain the frustrations expressed by some participants on the current void as they see it for pharmacy.

In describing the lack of representation, much of the focus was placed on the trade unions (IPU and HPAI), which currently were *perceived* as being focused purely on employment related matters. The membership of such unions is “*voluntary”* in nature and used primarily as vehicles for *“collective bargaining”* regarding work and economic matters.^35^^,^^36^ When the interviews turned to the regulator's (PSI's) current place, there was wide acceptance of independent regulation, something mirrored in most European and common law jurisdictions.^37^ The public protection rationale for this is outlined in the introduction, but there is still academic debate on the appropriateness of shared regulation, given professionals' adherence to ethical and altruistic codes of practice.^22^^,^^38^ A pertinent aspect considered was when the PSI on occasions diverges from its principle regulatory purpose: *“to protect…the safety of the public*.”^11^ This placed the PSI in a compromising position where it could be viewed as acting ultra vires (beyond the powers).^39^ Such examples of surrogate representation include speaking to the media, key stakeholders,^14^ drafting practice guidelines, and the provision of addiction and support services to registrants…etc.….^40^ The offending provision is s7 (2)(a)(viii) of the Pharmacy Act 2007:

*“it is the duty of the Society to take suitable action to improve the profession of pharmacy*.”^11^

When one compares s.7 of the Medical Practitioners Act 2007, there is no analogous role present therein.^41^ While this duty may have served a purpose during a period of absence of a professional representative arm, redesignation and delegation of roles is something which would need to be carefully considered between the regulator and any new entity going forward.

One issue which requires further attention according to the participants is who should have responsibility for oversight of CPD. This is not something unfamiliar in the literature and in fact the PSI commissioned a report on the matter in 2010 looking at other pharmacy jurisdictions and allied professions.^42^ The model which transpired was that which currently exists where the IIOP undertake responsibility for the *ePortfolio* and its review every five years.^19^^,^^20^ The hesitancy on the part of some participants about delegating this role is because of the *“regulatory requirement”* of CPD. This ensures life-long adherence on professionals to maintain competences throughout the entirety of their career rather than a simple validation upon initial registration.^43^ This again is something which will require further deliberation as to whether the PSI consider this something possible to outsource or does it fall within the legal confines of *delegatus non potest delegare* (“a delegate cannot delegate”).^44^^,^^45^ This question invariably brings one back to regulatory independence in that the *“arms-length”* approach between PSI and IIOP would not typically be considered appropriate for an independent professional body. This distance does provide the IIOP with a certain autonomy and safeguards to its members, in that the registrant has “absolute control” over their *ePortfolio,* and learning records are kept confidential and not disclosed to the PSI.^43^ In 2017, Crowe Horwarth carried out an independent review of the IIOP for the PSI, which has since informed the PSI's CPD model. The review praised the amount of progress the IIOP was able to achieve for CPD practice in Ireland in such little time, and also commended the “broad mission”, which enables and supports pharmacists in meeting the collective needs of patients. Going forward, Crowe Horwarth sought clarification regarding the precise role of this organization for pharmacy and its relationship with key stakeholders (e.g. PSI, HSE): “If the IIOP is seen as the *de facto* professional body of the future, then more resources and autonomy may need to be invested in it.”^21^ This uncertainty regarding roles was demonstrated by survey results among pharmacists (*n* = 365, only 39% viewing the IIOP as independent of the PSI, the remaining expressing views to the contrary or unsure).^21^ In reality, some participants did not consider it essential for a professional body to undertake the role of CPD, but rather focus on the other advocacy roles listed by participants. This is an approach similar to that which exists in Australia, where there is essentially three bodies: Pharmacy Board of Australia (*regulator*);^46^ Australian Pharmacy Council (*CPD administrator*);^47^ and the Pharmaceutical Society of Australia (*representative body*).^48^

There was a broad array of functions which interviewees felt could be addressed by this professional entity to showcase pharmacy and ensure it is developing on a par with.

other healthcare professionals. A few participants saw a role in lobbying for the appointment of a chief pharmacist in the DOH, which is a matter that has perplexed the profession since the post remained vacant as of 2013.^49^ This is to be contrasted with other professions such as medicine and nursing.^50^ While this is a somewhat tangential leadership problem, it was nonetheless considered important and related by some interviewees, especially if this entity intends to establish itself strategically to enable pharmacy to contribute to new integrated models of care in the health system.^34^

There was an array of interesting structural models being proposed by participants for an organization to be formed. Some applicants looked to other jurisdictions for inspiration namely the RPS model in the UK. This was cautioned by a few, and this varied view can be seen in the literature, with some considering it to be a forceful “voice” for all in the profession, while others have misgivings about underrepresentation and its convoluted organizational structure.^51^ Some of the more nuanced approaches, such as an umbrella organizations of the existing bodies, could be seen as reminiscent of the work of Canadian Pharmacists Association,^52^ the key distinction being the federal nature of that jurisdiction. Whether any of these models would gain widespread appeal is to be determined. In a similar fashion the fee structure would need detailed attention with a clearly prevailing view that membership fees should be non-negotiable.

One limitation of this study was the lack of consideration for undergraduate education. While it was accepted in general that roles of APPEL would fall within the confines of a professional body, not many participants delved into the detail of this any further, or indeed into issues relating to student membership. While our sample varied in terms of sectoral experience, all of our participants had a significant number of years of practice in pharmacy (+10 years) and thus we must acknowledge as a limitation that the voice of the newly-qualified pharmacist was not included in the study. Our sample included a slight majority of female participants (54%) which does not mirror exactly the gender ratio in the profession in Ireland (female to male pharmacists is 65%:35% (*per* 2018)).^53^ The findings have to be taken in context as well, as many of the cohort interviewed in this study were in non-patient facing sectors, and consequently may not directly reflect the wider population of pharmacists' views in Ireland on this matter. This is somewhat tempered by the fact that all of the pharmacists roles have applicability to patient care, and in fact four were in patient facing settings.

This study can be used as a reference going forward to inform decision makers in the profession of possible avenues to be explored in paving the way for the potential establishment of a professional entity. Future work could include larger scale studies in which the wider profession could be asked to consider the themes detailed and the proposed models in this study. While many issues were discussed there was a clear narrative emerging from the participants in favour of change, the manner in which that change will occur is an issue for the profession itself.

## 5 Conclusion

This study is the first opportunity provided for key stakeholders to give their perspective on an otherwise neglected topic, that of a united professional representative entity for pharmacy. The idea of professional representation has been seen as gap in the Irish sector by some academics since the advent of the new shared regulatory PSI in 2007.^14^^,^^22^ The findings give insight into the nature of the landscape, structural approaches and also some potential solutions to the financial and logistical hurdles policy makers may encounter when starting any new entity for the whole profession. Stakeholders drew inspiration from their own experience, but also referenced more robust models abroad in other jurisdictions (e.g. Canada and Australia), and other healthcare professions such as medicine and nursing.

When inertia and complacency become the norm, one is left with the unfortunate situation of accepting the *status quo*, without broadening ones ideas for development, improvement and progression. This paper provides an initial dialogue for a divergent pharmacy profession to reflect upon, and to consider how issues in contention may be addressed and unity of purpose achieved:

“*Alone we can do so little, together we can do so much*.” (*per Keller H*).^54^

## Declaration of Competing Interest

We have no conflicts of interest to disclose. The research received ethical approval from the relevant University Ethics Committee in November 2021. The detail of this process is available on request, if required.

## Figures and Tables

**Table:** 

No	Item	Guide question/description	Response
Personal characteristics			
1.	Interviewer/facilitator	Which author/s conducted the interview or focus group?	All interviews were conducted by POS.
2.	Credentials	What were the researcher's credentials (e.g. PhD, MD)?	BPharm, BCL, and LLM.
3.	Occupation	What was their occupation at the time of the study?	POS occupation at the time was a full-time final year MPharm student.
4.	Sex	Was the researcher male or female?	Male.
5.	Experience and training	What experience or training did the researcher have?	POS previously carried out research in other modules and in precious degrees. He was provided with qualitative research papers from his supervisor SM, who also provided guidance on using NVivo data coding.

Relationship with participants			
6.	Relationship established	Was a relationship established prior to study commencement?	No
7.	Participant knowledge of the interviewer	What did the participants know about the researcher (e.g. personal goals, reasons for doing the research)?	Name, stage of study and that the interviews were being carried out as part of completing POS's MPharm dissertation.
8.	Interviewer characteristics	What characteristics were reported about the interviewer/facilitator? (e.g. bias, assumptions, reasons and interests in the research topic)	Interviewees were informed via email invitation send from SM, that POS was carrying out this research to ascertain their views of key stakeholders on the proposed creation of a Professional Body for Pharmacy. It was also explained the manner in which the interviews would be conducted and recorded online via Microsoft Teams.

**Table:** 

No	Item	Guide question/description	Response
Theoretical framework			
9.	Methodological orientation and Theory	What methodological orientation was stated to underpin the study (e.g. grounded theory, discourse analysis, ethnography, phenomenology, content analysis)?	The six phases of Braun and Clarke's thematic analysis was used for analysis of the participant's transcripts. Rough codes were initially worked out, prior to more clearly defined ones being inserted into the NVivo software where a more comprehensive and relevant data set was obtained.

Participant selection			
10.	Sampling	How were participants selected (e.g. purposive, convenience, consecutive, snowball)?	Purposive sampling was initially used by identifying key stakeholders in the professional pharmacy sphere via their “Contact Us” section of their websites, e.g. IPU, IIOP, HPAI, HSE….etc… Following on from this a contact in the IIOP used their platform in snowball sampling manner to assist with further uptake in the study.
11.	Method of approach	How were participants approached (e.g. face-to-face, telephone, mail, email)?	Participants were approached via email invitation.
12.	Sample size	How many participants were in the study?	Thirteen.
13.	Nonparticipation	How many people refused to participate or dropped out? Reasons?	None.

Setting			
14.	Setting of data collection	Where was the data collected (e.g. home, clinic, workplace)?	All data was collected online via Microsoft Teams at a time convenient to the participants and location of their choosing, typically their workplace online.
15.	Presence of nonparticipants	Was anyone else present besides the participants and researchers?	No.
16.	Description of sample	What are the important characteristics of the sample (e.g. demographic data, date)?	All participants were registered pharmacists, seven female and six male. In terms of year's post-registration, eleven were > 20 years on the register, one was >15 years on the register, and one was >10 years on the register. Most of the pharmacists (nine) worked in non-patient facing representative or research sectors, such as HPAI, IIOP, IPU, UCC, TCD, HSE, Appel, TUH. While the remaining four also contributed to these sectors, they also had patient facing roles such as hospital or community pharmacy positions.

Data collection			
17.	Interview guide	Were questions, prompts, guides provided by the authors? Was it pilot tested?	Yes, they were./ Yes, it was.
18.	Repeat interviews	Were repeat interviews carried out? If yes, how many?	No, there wasn't.
19.	Audio/visual recording	Did the research use audio or visual recording to collect the data?	Both audio and video recording was collected via Microsoft Teams.
20.	Field notes	Were field notes made during and/or after the interview or focus group?	Yes, there were field notes.
21.	Duration	What was the duration of the interviews or focus group?	Average interview duration was 32 min (Range 16–80 min).
22.	Data saturation	Was data saturation discussed?	Yes, data saturation was reached around the 10–12 interview. The additional three interview was used as additional confirmational that no new themes were emerging in the dataset (*per* Francis Methodology).
23.	Transcripts returned	Were transcripts returned to participants for comment and/or correction?	Yes, all participants were offered a transcript for review. Seven participants did actually review their transcripts for comment.

**Table:** 

No	Item	Guide question/description	Response
Data analysis			
24.	Number of data coders	How many data coders coded the data?	One – POS, with input from SM.
25.	Description of the coding tree	Did authors provide a description of the coding tree?	Yes, a method of Braun and Clarke's thematic analysis to the transcripts is provided.
26.	Derivation of themes	Were themes identified in advance or derived from the data?	Themes were derived using both methods.
27.	Software	What software, if applicable, was used to manage the data?	NVivo version 1.6.1 (2022).
28.	Participant checking	Did participants provide feedback on the findings?	No.

Reporting			
29.	Quotations presented	Were participant quotations presented to illustrate the themes/findings? Was each quotation identified?	Yes, there were./Yes, there was.
30.	Data and findings consistent	Was there consistency between the data presented and the findings?	Yes.
31.	Clarity of major themes	Were major themes clearly presented in the findings?	Yes major themes are clearly presented in the results section.
32.	Clarity of minor themes	Is there a description of diverse cases or discussion of minor themes?	Yes, subthemes are presented in the results section under the appropriate major theme.

^⁎^Developed from: Tong, A. Sainsbury, P., and Craig, J. 2007. Consolidated criteria for reporting qualitative research (COREQ): A 32- item checklist for interviews and focus group. Int. J. Qual. Health Care 19: 349–357.

**Table:** 

Theme	Subtheme	Full quotat ions
1. Reflections on Current Professional Needs	Voids in Representative Landscape	*“We are the third largest professional collective after doctors and nurses. So to dilute our impact by having lots of different agendas in different bodies is a disaster**. (Participant 1)*** *“[A]t that time we needed to create a need at a national level, and create an agency that would produce that, and achieve that function in one of its primary roles, that was missed…..as a result all the other special interest groups…. all got their own role, but they're not there to represent the profession, they're not there to represent, to lobby on behalf of pharmacists.” **(Participant 2)*** *“They need to be independent of other factors, be that unions, commercial owners, all of these other aspects. **(Participant 2)*** *“And I, I believe it's negligent of all the various groups that mentioned, including the regulator to identify this is the knob of the problem here, we need to recognize that we're missing out on an independent voice of pharmacists in the country who are there simply representing the professional pharmacists.” **(Participant 2)*** *“I suppose it's interesting. I think we have to reflect on what we what we hope to achieve through the establishment of the IIOP and that we thought that would provide a leadership role for the profession. And I think we need to seriously reflect and see why that hasn't been achieved and realized. Not to make the same mistakes again**.” (Participant 3)*** *“[I]t's very easy to stand and criticise the status quo. Had the PSI not set up the Institute, not funded it in the way that it is, we wouldn't have had it and we wouldn't have done anything. The profession wasn't going to do anything, the health service wasn't going to do anything, the DoH wasn't going to do anything, the representative bodies weren't going to do anything, so the regular has stepped in and done a very, very good job…but it could be better.” **(Participant 1)*** *“[W]e don't have anybody that can do that [comment on professional issues]. So who do you hear on from pharmacy you'll hear the IPU, the HPAI virtually invisible, you don't ever hear from them; and PIER are kind of invisible because by the nature of PEAR… industry people, tend to….do their own stuff.” **(Participant 12)*** *“[P]harmacy just doesn't have someone and they've got kind of* ad hoc *representative bodies put forward but anybody who says that from the Union is kind of automatically you know there is kind of an element of distrust, which doesn't reflect on the Union. It it's just that they're saying, well, you know you're coming here to try and get the most money out of it for retail pharmacy, you know.” **(Participant 12)*** ***“**So if we look at what we have in Ireland, we have, the employee or trade union representation. That would be your HPAI for hospital pharmacy and the representation that they do to the health service is to advocate for employee conditions for hospital pharmacists. The IPU, the Irish Pharmaceutical Union also make representations to government and their mandate is to represent member interests with respect to the contract that we have for payment of services.” **(Participant 1).*** *“But none of those leverage the collective knowledge among pharmacists for the benefit of patients. And the fact that we don't have collective professional representation in pharmacy in Ireland represents a significant lack of delivery on our professional responsibility to society.” **(Participant 1).*** *“The IPU and the hospital pharmacists association whilst they both do some professional work, are of course ultimately there to represent their members concerning their terms and conditions of service essentially to the health service itself. So they are in that sense, whilst they may have other aspects to their nature, they are also trade representative bodies.” **(Participant 10)*** “*I think they[IPU and HPAI] have done their best to develop it….everybody is doing great work to make huge contributions, but I think it's very much almost on the side of what it could be. So if we had a professional body…they could… carry a lot more traction in their work.” **(Participant 11)*** *“Those unions cater to the Union membership. They don't cater to the wider population, from which their membership comes.” **(Participant 13)*** ***“**There are issues around a person's right to privacy around trade union membership and therefore….I think it is therefore a nonsense that a trade union body and a professional body would be one in the same, when by virtue of your membership to the professional body you are putting on a public badge to say that you are a member of the trade union, which should be protected information.”(**Participant 13)*** *“[A]s an example, if the HPAI, you know, speak and the voice that they represent is the voice of their membership, it is not for example the voice of hospital pharmacists and I think it's probably important to be watchful, you know, in terms of what, what proportion of the eligible population, in fact are members of that……I mean the IPU is first and foremost a what we would have spoken about earlier, a kind of a representation you know in in terms of I guess what you might call the labour rights and the commercial rights of its members, and yeah, so again, I think it doesn't have full coverage of all Community pharmacists of all retail pharmacists in Ireland.” **(Participant 13).*** *“But when we have members of the IPU it is a clear conflict, they don't represent pharmacist, they represent pharmacy owners. They are our unions and we don't want unions, we are a profession, we can have unions and you can be a member of a union if you want, and this is where we go back to recognizing that… mistakes were made and that mistakes need to be rectified, and that means it needs to be funded and needs what I would say it's a 10 year plan.” **(Participant 2)*** *“Like the IPU, very much does it on behalf of Community pharmacists, but they do a negotiation as well. We have the HPAI that advocates the role of the hospital pharmacists, but it's a unionized and union membership only…..I think it's definitely not fit for purpose. I actually think that they're all lobbying, and if you take the hospital, if you take the community, they are lobbying for their own interest groups only their own and their own membership, they're not lobbying for the profession, they're not lobbying as a as a professional organization**.”(Participant 3).*** *“I think the word patchwork is very accurate, and I don't think it's sufficient because I feel that there isn't a voice for pharmacy as a whole. There's the voice of the hospital pharmacists, as distinct from the voice of the community pharmacists, and then we have potentially PIER, which is the industrial pharmacists, and I just feel, wouldn't it be nice if we could just have a professional body where we all sign up and we say OK at some level we agreed that as a profession we have these value.” **(Participant 5)*** *“I don't think it's[the current representation] sufficient…. and I think it's very local, and it's very open to individuals as opposed to a profession.” **(Participant 5)*** *“Even though the IPU isn't a professional body, nor does it ever you know, sort of say it is, but I think that it could be one of those organizations that people think of because they do their CPD through that, or have done historically. And yet it is the voice more or less as far as I understand it of the owner of a community pharmacy rather than a community pharmacist.” **(Participant 5**)* *“I would completely agree with it. It is something that is badly lacking…. there is a massive void of leadership in pharmacy in Ireland. You basically have two union bodies, one the Irish Pharmacy Union, who are primarily made up of owners of community pharmacies or representatives of large chains, there is a small element of employee pharmacist input, but not really as much as is kind of owners and groups. And the other one is the hospital pharmacist association of Ireland, who are the Union for public hospital pharmacist really, and to the part that you have to be a member of the Fórsa Union, so another union I suppose.” **(Participant 7)*** *“I don't see them as being professional bodies, their unions and the purpose and role of a union is to negotiate on your terms and conditions your entitlements. The role and purpose of a personal body is to provide strategic development to a workforce that has a unique body of study, a unique knowledge base. And that's meant to be used for patient care. I think the IPU and the HPAI are excellent organizations, and they are ronseal, they do exactly what's on the tin, their unions. And they work really well and really effectively.” **(Participant 8)*** *“I think at the moment we're very split as a profession, so hospital pharmacists do their own thing. COVID has made that probably even worse. Community pharmacist do their own thing, we don't have a professional body to kind of pull this all together and say, let's look at this from the wider professional perspective.” **(Participant 12)*** *“I think there has been the evolution of pharmacy, if we could call it that, there has been patchwork exercise essentially. And the main component that the mistakes were made when the PSI became the regulator, it didn't identify a professional body in a role for professional body, and it retains some of the actions and so on and so forth. And yet it missed out on the opportunity because a professional body has to be able to influence and lobby and represents professional pharmacists in an unbiased fashion.” **(Participant 2)*** *“And then the void was left[after the Pharmacy Act 2007] and…there was nobody left, so I think if you were community pharmacist, you had the IPU, but the IPU had always been there, but everybody else had nothing else.” **(Participant 9)***
	Public Benefit	*“[P]harmacists do an awful lot…. that's not seen…..we've evolved out of dispensaries, and I think we're so much more than that now…..it's really important that we…start to…. show our transparency and what we do as a profession for the public first and foremost, and also for the profession.” **(Participant 11)*** *“I think it's a significant gap in the Irish health care environment that has repercussions and consequences for patients. I am a hospital pharmacist, my practice is about patient care. I have a unique body of knowledge and skills that enhance patient care. And I would see that a professional representative body, and I don't know if you're going to ask me this question further in, is about the strategic development of my profession with the objective of enhancing the patient care. Because if you develop pharmacy beyond our legislative and regulatory role, you increased our pharmacy practice. Our scope of practice and it becomes less about an individual and more about a professional and a workforce, a strategic approach to how a workforce care for patients.” (**Participant 8)*** *“In setting itself up as a profession it commits to using that knowledge for societal benefit rather than self-interest. So the importance of having professional representation is bringing to bear a collective responsibility for society to use the knowledge across the profession for the benefit of patients in this case. And the lack of professional representation means that what you're left with is representation on different agendas.” **(Participant 1)***
	Underutilisation of Pharmacist skill-sets	*“I don't know some element because I always think that Irish people are very keen to, you know pick up the baton and change and you know I think we've seen a lot of that in the last year. You know pharmacists vaccinating* etc. *What else, you know, pharmacist prescribing like to I suppose be part of new initiatives to say, well, you know, yes we can and you know to focus on the knowledge, the skills and the competencies that pharmacists have and say you know, especially given, and to feed into what you know the government are saying. The government is saying, you know we need to do more with less or more with the same. And I see pharmacists have been pivotal to that, they're already* in situ*, they're highly trained, they're highly skilled, a lot of them are doing jobs that they could do, you know quite easily without breaking a sweat, and yet they're not being used to their fullest potential. And that's not about using them to get the most out of them, that's also increasing job satisfaction, because I'd also be interested in this professional body to do a kind of a scoping review on you know where are pharmacists working, so that we could also do some workforce planning going forward.” **(Participant 5)*** *“[A] professional body could really start driving that on and then actually looking at the jobs that pharmacist are actually doing in community pharmacy, and saying right does a pharmacist actually have to do this or could an accuracy checking technician do it, like they have in the UK and actually what is the best use of skills both for pharmacist and for the technician.” **(Participant 7)*** *“Pharmacists tends to be reasonably clever people, and quite empathetic people, so I think actually being able to use the skills rather than being surrounded by layers and layers of I can't. Simple things that that would actually really helpful to retention**.**”**(Participant 7)*** *“I think there is a space and the PSI is my regulator and I think there is a space where an organization that has the strategic workforce planning piece, that has the vision for the profession that lobby the Department of Health for us to get a chief pharmacist. So there's some strategy coming into the health service around pharmacists, and how pharmacists can improve the health service and pharmacists work a lot at the transition of care, we're experts at the transition of care, so, and that expertise isn't in any way adding to the debate or enhancing services for patients, beyond the immediate individual patient. And yet all that knowledge and experience could and should be involved in the strategic stuff within the health service, and until yesterday I would have said that wasn't happening.” **(Participant 8)*** *“We have a workforce that is not in any way being developed. There isn't workforce planning unit for pharmacy in the HSE. There isn't a pharmacy strategy development in the DOH, and there is no organization out there at the moment that is looking at workforce development with the patient at the core of that interest” **(Participant 1)*** *“So we have a model of care, national model of care for stroke, with no mention of medicines management or reconciliation or optimization or anything by pharmacists. And we have a national model of care for older persons, which has just recruited 460 additional health care workers with ambulatory care hubs and frailty at the front door hubs across each of the CHO areas and not a single pharmacist. Knowing that the WHO patient safety agenda at the moment is looking at polypharmacy transitions of care and high risk situations and there's no pharmacist. It's not because nobody understands the role of pharmacists, it is because there's an ignorance: one there's no pharmacist in there to advocate or to inform, and there and there's a perception that advanced nurse practitioners can do this work……And so people don't think that they're missing medicines management, they think that's being done by the clinicians, but the only clinician that's capable of doing a medicines management on par with a pharmacist is a geriatrician, they're very good at it. Otherwise, everybody is very specialized and advanced nurse practitioners are specialized, they're wonderful in their area, but nobody has the generalist knowledge that a pharmacist can bring to look across the piste and reconcile medicines across multiple prescribers and there are multiple systematic reviews that demonstrate that.” **(Participant 1)*** *“Yep. Because But we are some of the brightest people like I, I have pharmacists working with me, I always think, they could have chosen any profession and they could have been successful in any profession, but they chose pharmacy and we underutilize pharmacists. You know there is a legislative requirement to what we do, but it is only one domain of practice. But because our regulator regulates us and does our educational training against one domain of practice that's kind of what comes through. And it would be wonderful if there was a body who are responsible for the vision. Because the PSI can't do that because there our regulator. No matter how good they are and their super at what they do, their hands are tied. They are not constituted to be the visionary leaders and your professional body should be.” **(Participant 8)***

Theme	Subtheme	Full quotations
2. Regulation	Independence from the regulator	*“I feel it's negligent in the Irish situation that we don't have a professional representation body, that's distinct from the regulatory body**.” (Participant 2)*** *“The regulator is not a representative, but is there to protect the public interest by applying regulations to ensure consistency of service delivery.” **(Participant 1).*** *“Separate, I think it's very important to have, it is almost like church and state, I just think they have to be completely separate, otherwise there are obvious conflicts. And you know they have, it's not like they're working towards different goals, but I think the water can get muddied somewhat, because regulation is pretty much black and white and the profession is not black and white. It's made up of people and it's made it, you know.” **(Participant 5)*** *“The only way in which a professional body could be credible would be if it were created independently of the regulator.” (**Participant 10)*** *“And I think there should be complete separation from the two in the same way that like an Garda Síochána have separation of the Garda Ombudsman for the kind of policing of the police force, there should be separation of the PSI from any many proposed professional body.” **(Participant 7)*** *“And whilst the arm's length thing kind of works. It's only at arm's length, there is still input. I mean the PSI goes to the Department of Health and tells them what they're doing. So this is what we plan to do. This is our business plan for the next five years or whatever. And this is how we see this. And if somebody in the department I am not saying that they do, or that they would, or that they would have any ill intention. But if somebody in the department says, well, look, you know, I mean, why on Earth are you doing that? Why you're not doing something like this? It is simply that it's not appropriate” **(Participant 10)*** *“The IIOP are essentially fed their funding based on delivering exactly what the PSI tell them they want them to deliver, so there's no autonomy in what they do. There is a very clear expectation that they do everything exactly as set out in the contract, and so the level of control over the IIOP from the PSI is unhealthy if the IIOP is to expand its role in support of a professional agenda.” **(Participant 1)*** *“But notwithstanding the ambitions for that and the stated ambitions, you have to go back to the core operational aspects, which is ultimately it [the IIOP] was funded by the regulator and therefore whether you like it or not, the agenda is set by the paymasters, which is the regulator.” **(Participant 1).*** *“Completely, absolutely separate, like at the moment. For example, the Institute of Pharmacy is controlled and owned by the Pharmaceutical Society of Ireland. It should be separate from them.” **(Participant 12).*** *“Yes, I think they are two distinct you know….So there are two distinct functions that obviously the PSI is it as it exists that their function is to protect the public and I mean, I do appreciate that they do take on projects that support the development of pharmacists and pharmacy practice, but ultimately I think you know their primary function is to support or to protect the public and ensure that standards are upheld. And you know. So I think separation of the two is a good idea.” (**Participant 6).*** *“Yes. I think that such an organization or a platform would have to be independent of the regulator, but equally would have to have a relationship with the regulator.” **(Participant 4)*** *“[T]he regulator needs a professional body for independence, and the professional body needs independence from the regulator. So I can't see at all in an effective model and any of the models I've seen it, it is a natural progression that the two would become standalone, independent agencies. They might have been the case back when I was graduating, that the same body, but in terms of the primary roles to protect the patients, and to represent the pharmacists and best practice the two of those need to be independent as I see it.” (**Participant 2).*** *“And so obviously you need to have professional regulation as well to protect the public because, it's one of the oldest tropes in the world that come professions are a conspiracy against the laity.” **(Participant 12).*** *“And the authority is the PSI, the PSI approves what they do. You know the governance structure is they answer to the PSI. You'd like to see them answering to a professional body.” **(Participant 8)*** *“where matters of the profession, are dealt with as distinct from matters of regulation. People tend to confuse or synonymous the two and that is a problem. I've heard people say ‘Oh well, you know the PSI is a professional body.’ It's not, it's the regulator, their mandate is very different to what I think a professional body could and should be” **(Participant 5)*** *“A clear example of that was the devolution in the UK of the Royal Pharmaceutical Society of Great Britain into their General Pharmaceutical Council, which is regulatory and the Royal Pharmaceutical Society which is the professional body, and I think that model seems to work well.” (**Participant 5)*** *“I think it's very, very important that they have a degree of autonomy as well as oversight. So yes, I think they should be independent, but collaborate together for the common good.” (**Participant 11)*** *“I think they should be independent, but I mean they have to be able to talk. You know, there might be someone on PSI board and someone from the PSI on this board, because I think we have to stop treating the PSI as the devil incarnate, and realize you know they have a role to play and that role is to ensure we are the best pharmacists that we can possibly be and …to… protect us…..indirectly from ourselves.” **(Participant 9)*** *“I don't think, personally I don't think it has to be completely separate. I think there could be two arms of the PSI, one to do with advocacy and one to do with regulation. I can see though, where I can see where the waters could get muddied. In that the PSI if there was a conflict of interest. Yes, it's better to have them at arm's length, but they actually do need to be talking to each other as well.” **(Participant 3)*** *“So the IIOP always maintained that there at arms-length and I do believe that they are independent, but the PSI still require them to report every year on the people have submitted their portfolios that they're keeping that up. Like absolutely, there is an obligation as a registered pharmacist to keep our registration up to date, and to keep our knowledge content up to date. I'm not sure that we need this arms-length, I actually think they could both be housed in the same building. But I still think you can keep separation, but still have an over-arch……like then I'm coming back to the Nursing and Midwifery Board, how are they managing it, they are in one organization, but two separate….we have to trust ourselves and enable ourselves to do this. And I think as members of a profession, if we're not achieving or if particulars aren't achieved, we should be willing to call that out.” **(Participant 3)*** *“Then there is new services, and I think the society has done these things well, for example, you know vaccination services, extremely well done by the society, but it's not really their role like should that not be done by a professional body? You know at the regulator report part of it is the society but the standards you know is that really necessary for that to be done by the society**.” (Participant 12)*** *“The PSI has originally had that role, and while they do have a conduit to department which is essential. They're conflicted when they're asked to make representation and they don't do it, and rightly so, they can't do that because they can't represent the opinions.” **(Participant 2)*** *“I think there is overseeing and then there's regulation. I think their structures would need to have very clear delineation in terms of what their actual roles are. So I think we can't have a professional body without a regulator, but similarly I don't think we should have a regulator without a professional body, and I think the two need to dovetail, but they need to have very distinct roles. So obviously the profession has to be regulated, because it's a very patient-centred profession. And it has to be regulated and there has to be safety parameters there for patients, that's really important in terms of you know competency to practice, negligence* etc. *But they're also has to be the autonomy for professional body to act as a professional body and then the regulator to regulate. So I think I think they'll always be intrinsically linked and collaborate as they should, but I think they have to have autonomy and they have to have very clear role description.” **(Participant 11)*** *“So I feel that they have to be independent, the role of the regulator is there to protect the patient that is their number one priority. And in addition, I would suggest that the successful models of professional groups and regulations have to be independent, so my feeling is the regulatory bodies needed to leave it to the professional bodies to write and draft best practice guidelines, expectations and create special interest groups so it shouldn't be the regulator defining the regulations, which is really not how I believe it should operate. There should be an independent agency or group which are drafting regulatory guidelines, inspection guidelines and all of these other factors. And yes, of course the regulator can have oversight and transcribe those into regulatory standards, but there has to be an element where the profession can feed into regulatory guidelines**.”(Participant 2)*** *“I don't think that the PSI and the…………the legislation that sets up the PSI is and it always sounds patronizing to say so, but it is in a sense, an Irish solution to an Irish problem. The Minister who set it up didn't like professionals. She was of the view that all professionals are in essence a conspiracy against the public right, which is essentially a view of George Bernard Shaw's and that therefore she didn't want there to be really a professional body. So she created a regulator, however, it was apparent I suspect, to a lot of the civil servants or the public servants that in actual fact, if pharmacy was to develop it, it needed to have the capacity to do so. So in that legislation is inserted the clause that the Society has in actual fact a responsibility to help advance the profession or whatever it is, the exact wording is. But that isn't the function of a regulator, right. If you look at the background to the to the Medical Council of Ireland, it doesn't have a role to advance the medical profession, it is a regulator. And sometimes it forgets that and says things that it shouldn't say in public really. It speaks out on behalf of doctors, well, in actual fact it's really there as a regulator. And in my view, the regulator should just regulate, so therefore there shouldn't be any connection between the professional body and the regulator, otherwise the regulator is no longer independent. We have the unusual circumstance in which the regulator still has a role in developing the profession. In truth, it really shouldn't have it. However, we're stuck with it, so that's one of the reasons why the PSI does some of the things that it does.” **(Participant 10)*** *“And I do think the regulator, you know they're there to regulate and they need to be doing that and it's hard, you know, they're probably you know, between a rock and a hard place at the moment, because people kind of expect them to represent, but they're like that's not a job. And I think it would actually really help the PSI because it would be easier for them to clarify their role with the HSE and with representative bodies and with stakeholders. If it was like no, no, you need to go to that crowd over there for that stuff, and we'll deal with this stuff. Whereas at the moment you know they're probably under a bit of pressure to be doing a little bit of representation in the HSE, because nobody else is doing it and that's the juxtaposition between the two. You know that kind of way, so I think it will be helpful for the PSI to have a body basically.” **(Participant 9)***
	Educational Accreditation	*“I really do think that there would need to be some consideration of where CPD sits and continued accreditation.” **(Participant 1)*** *“Where does as you said are IIOP belongs somewhere, but the society might say that belongs to us because it's a mandatory thing. And maybe they want that and fair enough then if it is, but where do you go after that. Like what's the postgraduate stuff on that you know?” **(Participant 12)*** *“But the role of the IIOP in terms of CPD, in terms of accreditation of courses, in terms of putting mechanisms in place for a portfolio, and that would have all formed within a professional bodies remit, similar to one as I understand it is done for RPS and other professional bodies” (**Participant 2)*** *“OK at some level we agreed that as a profession we have these values. These aspirations, these missions. And absolutely there can be all those special interest groups or you know specializations or credentialing or whatever the case may be associated with that, but again, credentialing is more regulatory thing.” **(Participant 5)*** *“If what we want is good quality accredited undergraduate programmes, and good quality accredited ongoing education, then you think, OK, well, how do we achieve that? If that's within the PSI, that's fine, if it's not, that's fine**.” (Participant 1)*** *“So you know, this is what I mean about places like universities, places like regulators, they don't understand practice. And so would a body like the PSI regulate, yeah they could they could regulate all the education or they could assign the responsibility for that to a professional body and in most places in the world, no not most, in many places in the world a good relationship and good alignment between the professional body and the regulator is always very good. And if the regulator can entrust some aspects of accreditation to a professional body, I think that represents a very good ecosystem within the profession, provided that the appropriate assurances and checks and measures are in place to assure quality at the end of the day.” **(Participant 1)*** *“It could fit into a professional body role, it doesn't have to. I mean there is probably just about enough separation between the PSI and the IIOP to enable the IIOP to function. It's not an ideal circumstance, but then, it wouldn't be necessary if the IIOP were separate from the PSI for it to actually be associated with the professional body. It could exist as a separate body itself. So there are pros and cons to it being where it is, there are pros and cons to it being attached to a professional body and there are also pros and cons to it being an independent body in its own right. I think the professional body, whilst it would have views about continuing professional development and so on, doesn't necessarily need to administer that, or doesn't necessarily need to take on that function in order to be able to express those views and to influence what happens.” **(Participant 10)*** *“So I think from an education point of view I don't see that that's the role of the regulator. I'd say that very much….and continuing development very much the role of the professional leadership body. And I think they could provide frameworks and structures that can feed into and like career credentialing and portfolios etc that the regulator could use as a tool if you like in terms of assessments and regulations, you know, if they're called to do so….or if they're doing whatever you know they might be where they're actually carrying out a review of a particular pharmacist for whatever reason. I think one can certainly feed into the other, but they have to have autonomy to be either a regulator or professional body.” **(Participant 11)*** *“So just to go back to one point about the CPD, like CPD can be voluntary. So many people as part of the system and the IIOP so you can generate portfolios, you can build your content and you might use it as your own portfolio and it's your property or you keep it within the PSI mechanism. Not every one of those needs to be accredited, not every one of them….so it's only if you want to practice does your portfolio get reviewed by the other, so ultimately there may be many pharmacists who need CPD for own personal reasons, but that their portfolio it's maintained through that system, and at one stage it only become, and it may not need to be accredited. So I don't see the professional body as self-accrediting content. I don't know how IIOP do it to be honest, but it's really a self-reporting portfolio and a mechanism for generating CPD and recording CPD. Accrediting and assessing that should only be if you need to, and it should be then transported, your portfolio should be shipped over. And my understanding is this, why it might have been contacted, my understanding is even still it would be a fault of the regulator to expect that you can outsource that to another body. They are two very different things assessing standards and providing that, you know you can't this is the complication as I see it.” **(Participant 2)*** *“So I think for the undergraduate I think, I think there should so on the accreditation panels. I think there should be a representative from the PSI and from the professional body. I think it should be a dual process. For CPD, I actually think that it should go to the professional body now, if that CPD leads to expanding roles or separate register entitlements, there has to be a regulatory involvement to it, there has to be a set of criteria and standards. But I suppose the Irish Nursing and Midwifery Board have kept them both simultaneously together. And they've managed to do it without separating them.” **(Participant 3)*** *“Yeah, well, the legislation requires that it's the PSI that accredits, but when the PSI goes to do that accreditation, they probably have to bring in outside expertise and then they bring in that expertise which reports to the PSI and then it becomes the PSI's accreditation. That's it, if there was a professional body that had that expertise, then certainly the PSI could outsource it.” **(Participant 4)*** *“I think that ultimately the regulator has to ensure that, well, they are the accrediting body, and as such at the moment I don't see any wiggle room in that the way that things are currently structured. Like they are the accrediting body end of. But going forward, I don't know is my honest answer. It would depend on whether this professional body is, you know funded appropriately, whether you know the expertise are present and also, then I suppose if they are going to be the accrediting body for you mean for somebody to enter the register and to maintain their presence on the register or?” **(Participant 5)*** *“Well the IIOP do a lot of the delivery of PSI elements of the CPD elements, and like you logging your CPD with IIOP ultimately, which I suppose kind of the PSI do the policing of that. Which seems reasonable thing, and I'm not sure……..So the CPD, like delivery of the CPD by a professional body, would be entirely reasonable, the policing of it by the PSI would be entirely reasonable. So obviously there would have to be a conduit between professional body and the PSI.” **(Participant 7)*** *“So yeah, I mean, I think it could definitely be incorporated into it because I like knowing in the background that the PSI want to bring the CPD piece back in house to a certain degree, I think there's elements of what the IIOP, like that could be split in two, the IIOP functionality, do you know that kind way. As in the piece where they do the assessments and the reviews could all be done by the regulator, but the piece where they do the webinars etc could be done by this organizational body and then that could be shared with the IIOP or take it off the IIOP, so that there is often a crossover between the two.” (**Participant 9)*** *“No, a professional body shouldn't be doing that. It should be providing a framework, it should be providing guidelines, it should be doing that. But the actual accreditation and checking of this should be as I see it, a role of the of the regulator.” **(Participant 2)***

Theme	Subtheme	Full quotations
3. Structure and Functions	Structures	***(1.)“***Yeah, I mean, yeah, I suppose the institute of pharmacy practice, but the only thing is, I think you should just start with a clean slate is the honest answer. Because if you bring in order organizations, they come with their politics and they come with their agenda and they won't be seen as open to everyone. So you know anyone who doesn't do CPD then would have no interest in the IIOP, you know that has a singular function, which is professional development and that's what they do and they do it really well, you know. And I think the hospital crowd or I think the IPU with the community crowd, PIER or that everyone else who is lost in the void. And then, and even at that it's generally industry, you know and a few academics, but you know all the other people who might be in management…. there's more to being a pharmacist then just the obvious roles and there is loads more you can do, and I'd love to get those guys back into the pot. So I think a blank canvas for me would be the way to go because then it has no history. Yes, start afresh I think would be the simplest, cleanest and probably you get more buy in.”**(Participant 9)** “And so you know, my sense is I am a great believer in a clean slate and start from fresh because sometimes you have legacy issues where, we've always done it this way and, well if we are not going to do it this way, then I don't know, or vice versa, when we've never done it this way, so why would we do it this way? Whereas I'm kind of a believer in, well, you know who feels like they want this? I mean, I think it would take a lot of effort.” **(Participant 5)** Realistically the funding that would be required to create such a thing; and seismic to me would be create a body in Dublin which might be just an office of three or four people who have a reporting line to an administer, who has the ability to confer a stamp so that people would like to be membership of it.” **(Participant 2)** “So I was going to say whether or not, is there a role for professional body, arguably yes. Is there a space for professional representative body like that? I'm not sure and there's a politician from 20 years ago called Michael McDowell, and he always said “it's one thing to say that there's a niche in the market, but you have to ask if there's a market in the niche. And if there's to be another body representing pharmacists, then that body will need to be funded and I don't know that pharmacists are ready to fund yet another organization. There already funding the PSI, the IIOP, the IPU.” **(Participant 4).** “I think having an independent body for pharmacy here to steer that would be a lot better approach rather than like say that existing organizations which you know some of them have maybe damaged relationships with other stakeholders in their sectors. I think really a carte blanche, is the way forward here.” **(Participant 7)** ***(2.)“**Yep, absolutely, and again I think the IIOP do a stellar job at education and therefore I think that that's why I would see, you know, probably the best fit of this support body being in conjunction with the IIOP. So there could be complementarity around what is going on there, you know, and that there could be a needs assessment and that they could then be complementary delivery of additional material. For example material you know that for a variety of reasons may not…. you know that the IIOP may not currently have the freedom to deliver.” **(Participant 13)*** *“I would agree only if IIOP could be completely separated as an independent entity, so you would have to create….. some sort of separation process, create a new company and give those roles to it.” **(Participant 12)*** *“I think we probably gave insufficient time to the importance of the environment being right for a professional body. So the convenient thing to do at the moment within pharmacy would be to take one of the existing bodies and say, actually you can do the work, saves us a lot of hassle in setting something else new. But the agenda isn't going to be driven by the right people, there's no, there's no agenda at the moment being driven just by pharmacy professional interest. And then, if we did get it set up, the system isn't ready, pharmacists are not being proactive in contributing to consultations or in shaping their health service.” **(Participant 1)*** *“I've given it a huge amount of thought and I've gone through the process of, ‘well, the IIOP could be the professional body and wouldn't it be fine, everything would be sorted if we just had funding and everything would be grand if we had a professional representation.’ And the more I see, the more I'm going ‘oh my God, I've managed to get into the HSE. I've managed to say let's get pharmacists into the models of care. I've managed to get them onto those tables’ and even if you have pharmacists included in the models of care at a national agenda; you then are relying on your colleagues working in Community and working in practice to step up to the plate and deliver those services and not to do brinkmanship and stand off and say ‘we won't do them unless you give us a career structure or you reward us for tenure, you pay us this, you do that in the contract.’ You, you know, even if you had a professional body, you then would be reliant on your colleagues across the representative organization to take more of a professional view, so you know, whoever wherever this body sets up, those are the skills that are needed, not academic prowess, not, you, know, convenience of what's already there. And that's a big ask. This is like a multidecade project.” **(Participant 1)*** *Use of the IIOP - “Yes so that would need to be looked after funding because you know, are you going to bite the hand that feeds you? Probably not but I think like if you were able to, sort out that funding then I think yes the IIOP, there is a lot of good people in the IIOP, I think it has got good leadership. I think that could be version 1.1. of the body, and like it exists and it's kinda doing what we kind of want it to do, but just it would need to grow a little bit more and bit tricky on the funding piece like. I mean is it funded by members you know. So if it's funded by members, what do they get in return, would that fee be maybe be tax deductible? You know there's a lot of pieces around that the funding part but, yes I wouldn't have any issues at all with the IIOP starting the version 1.1 of it. Even the name Irish Institute of Pharmacy, it is a reasonable title, like it kind of does exactly what it says on the tin and so something like that will be will be totally fine I think.” **(Participant 7)*** *“I think the Irish Institute of Pharmacy has been given the task around the education piece. I would love to see the competency framework expanded beyond this initial. You know the current one is for the first three years post registration and I'm more like 23 years on the register, you know. So It would be wonderful and for that part of the IIOP to grow and at the moment how their areas of responsibility and how they are constituted, they would need to be changed and I don't know. Sometimes I mean there were relatively new organization so but the culture organization has set by that founding piece. So absolutely it's a structure that could grow, but it would, it would change what the IIOP is, and there are significant additional domains there would need to be given to it and it's the organization would be very different and if it was an organization that was interested in that, super, but it's always hard for established organizations to transition and change. It's not impossible, absolutely not and as an educational training space, you'd expect them to be able to do that to be agile and to be able to move but that would be for them to reflect on, I think and to consider. But it would change, I mean their constitution, wouldn't be the IIOP as we know them today.” (**Participant 8)*** *“That would absolutely be a very short term, convenient way of approaching it, but completely the wrong thing to do. We have a professional need, what needs to be done to service it? Not we have a body[IIOP], how can we retrofit it to deliver on a professional need?” **(Participant 1)*** *“If the IIOP was the way to do it and if pharmacists were willing to put some, you know they would need to contribute financially. And if the PSI changed the model by which they allocate the funding because they could, they could say we'll allocate funding for specific services around CPD, e-portfolio, −e-portfolio review, practice review and we'll allocate you a certain amount of money, and you have to show us that you're delivering value for that, but we're not going to dictate what it is. They could do that and the DoH who are currently partially funding the IIOP could do likewise, they could say we're going to give you some money, because those two funding pots are already there. And the members would need to step up and say “OK, we'll get our basic e-portfolios CPD stuff out of the institute, but if we want more, if we want coaching, if we want development, if we want career structure, if you want advanced practice, if we want advanced competency frameworks, we will pay for them. If we want to be recognized as fellowships.” So I think that's a model that could work, I think the IIOP, now I am entirely biased, but I do think the Irish Institute of Pharmacy has demonstrated its credibility to hold the tension between the profession and the regulator, and has enabled six and a half thousand pharmacists to adapt to an entirely new CPD model, and be subjected to practice review with absolutely no turmoil. And it's done in a respectful and collegial and collaborative and peer led way. So I do think the Institute is the best bet, but it would need to change, it would need to change the service level agreement it has with the PSI and they'd have to take their hands off it. It would need the money to come directly from the DoH and not be channelled through the PSI and it would need the profession to also fund it, and it would need to have the current staffing maintained.” **(Participant 1)*** *“Yeah. I think the IIOP, if it was so decided, I mean, it's obviously needs, you know, focus groups and input from so many different people and bodies. But I think the IIOP have developed a huge experience of the profession, they communicate so well with the profession. They have obviously got a wealth of information in terms of webinars and courses, they have been there from the start in terms of the development process and there's a wealth of information. They provide such a space for the profession, they're so positive and instructive. I think if they could certainly transition to being, you know, the basis for the professional body. I think it will be just a shame to say, well you know, we won't have the IIOP anymore, we'll start again. There's a wealth of experience and learning, and that's taken years to get there for them. And they are so constructive about learning from that kind of reflexivity of response. So I think that just really needs to be captured all the work that they've done, and all the energy they've committed to that. So I, I think there will be very well placed if that's what came out of, you know, a consultation process to move from being CPD to a full professional body.” **(Participant 11)*** *“I can tease out the two options, so I guess we've got a regulatory body, and for good reason, and they, you know are at arms distance, or whatever phrase you might like to use from the development body. And we have the IIOP which in some ways and you know their remit is to support pharmacy development, although that being you know largely constrained by the current legal set up for you know that that governs pharmacy practice in Ireland. If I think about the UK and I guess I am thinking about, you know the genesis there being that there was a you know where the regulatory body would have had some degree of development work and then there was the dissociation, you know between that and the development body. So I think that there is the potential for the IIOP to be the body, if it were to be further resourced you know and if it's remit was broadened. And I don't know I'll be honest, the intricacies of the legalities behind it, I don't know, but I'm not sure that it would make sense to have yet another body, you know, so our regulatory body, the IIOP and then yet another body. You know when I would wonder about the interactions between all of them and that their potential you know for overlap, and just how they might work meaningfully together you know. So I think what I might foresee would be expansion of the resourcing and the remit of the IIOP.” (**Participant 13)*** *“I would disband the IIOP, I personally think it needs to be……I don't think the IIOP has generated or has delivered for the profession to what it originally set out to deliver. And it has developed the platform for lobbying and maintaining our CPD and doing our critical evaluation, it has done that very effectively, but has it lobbied and has it influenced on behalf of pharmacists? No it hasn't and maybe they would argue that hasn't been their role or the remit, but we all have as a member of the profession, we all have a role to advocate and to lobby for their profession and I think the IIOP, I think it would be a rebranding, I think it would be a seismic shift of what that is.” **(Participant 3)*** ***(3.)**“But they could be an umbrella organization, so essentially the only way the only solution is I see it now is, that you allow each of those their identity, but they're affiliated under an umbrella organization, which is a bit like how the European Medicines Agency work, that you have all the various agencies and groups and special interest groups working within that, but they all would have to sign up to a kind of a memorandum of agreement to say we would have this body representing us at a top level and so on. So the only pathway as I see it going forward is that we have missed the opportunity of creating a professional body. and we are up against trying to recreate that central hub that has a central hub of excellence to be able to influence, or at least make representation in an unbiased, objectively independent of commercial, because this is not an IPU, that obviously is a serious conflict. We are simply representing the profession of pharmacy and providing pharmaceutical knowledge on behalf of a profession, they need to be independent body and they need to be independent of other factors, be that unions, commercial owners, all of these other aspects….And each pharmacist has one vote, is how it operates and that is not how the IPU is set up.” **(Participant 2)*** *“So to answer your question, could we foresee a standalone body? No it is too late, and secondly, even a standalone single entity would just get too complicated given that it's a very diverse flock within pharmacy, so that that doesn't serve its purpose. Could we come up with a system where we have all of these other bodies have evolved, and where the only way I could see it is that you would develop a type of memorandum of understanding that over the next seven years each of these organizations would agree to work with another body, another agency that's created that is that knob that connects and provides the independent voice, but that has to be funded. And so you establish a memorandum of understanding and in over a number of years and the different groups will either merge or co-fund and maybe developing memorandum of agreement into a memorandum of understanding. And then you would have representation from those, so it's a bit like merging of those entities. Now each of them will want to keep their own identity as a special interest group, but there would have to be pooling of some resources. Now as a financial model, if I was a business person trying to develop a new professional body that would generate its own income stream, that would be self-funding over years, this is not the way I would go**.” (Participant 2)*** *“So the assumption that a single professional body will keep a very diverse flock together under one organization and expect them to pay fees even though they don't really see the need, I don't think that model would work forever, so I think it's unrealistic or indeed aspirational to assume that a new body could do all but the other groups are doing. So that's not really going to work and in fact, the limited example and it's only one in the UK, and my understanding is that even now they're beginning to splinter….. but give the title PSI to a new agency that's created and think small and let that agency grow and then agree that…” **(Participant 2)*** *I think pharmacy representation would benefit if there was a higher level forum, like a Pharmacy forum where all of those organizations would come together. So we're not talking necessarily about a new organization we're talking about a forum or a mechanism for bringing them together and to ensure that areas of common interest can be identified, strategic approaches to achieving common objectives can be taken, and this if there are areas of difference that those could potentially be ironed out…. The reason that I'm talking about something other than a professional body is what I've seen in other countries…..what I see in professional bodies is to become very academic. They've become as in the structures, the governance, the directors, the leadership, they're all pharmacists in academia, and they can become a bit divorced from the realities of practicing pharmacy, whether it's in a hospital or in a community pharmacy, and they become a little bit out of touch. And if that happens then they can do great work, but that work becomes irrelevant to the pharmacists that are actually trying to do a job….It would be a collective, so an analogy would be the Irish Interprofessional Association and that's not something you're going to find it easy to look up, but that's an association that brings together representative bodies and professional bodies for the liberal professions. So The Law Society, The Bar of Ireland, Tax institute, certified public accountants, Engineers, Ireland and the IPU for Pharmacists, Dental Association, medical organizations, we're all in it. And it doesn't have a secretariat of its own, it has a rotating chairmanship and then whoever is the chairman of it provides a secretarial and the logistic and the infrastructure. So that's the kind of platform I was thinking of and then it would be the hospital. Pharmacists PIER, the IPU and whomever, whatever other group will be part of it, could rotate the leadership with that organization and could provide the secretarial assistance and support and the administrative support that's needed. Medicines for Ireland is a trade association representing the manufacturers of generic and biosimilar medicines, they operate on a similar model, whereas the research based Pharmaceutical industry has a professional representative body called IPHA… I don't think there should be a fee because I don't think it would have individual membership, but I'm talking about is a different model. It's closer to the Canadian Pharmacists Association, the members in the Canadian Pharmacists Association are the provincial pharmacy associations. So you're a member of your own association and then your own association is a member of this umbrella body. So I'm thinking in terms of an umbrella leadership body rather than a new organization…..It would start small, grow organically, and start as a cooperation and collaboration between the representative bodies of the different sectors of the pharmacy profession. Would it grow into something in future if it's working, yes, it would, if it's not working then it won't….“In the UK years ago, they had a group called Pharmacy Voice and it was doing something similar, it tried to bring together all the disparate pharmacy representative groups in the UK as an umbrella to have a single voice, it only lasted a few years because there was the various members within it fell out with each other.” **(Participant 4)*** *“You can have a forum. The difficulty is that, as I say, the IPU and the HPAI apart from their professional activities, are also representing the needs of their members in terms of their terms of service. And the IPU already has quite a difficult balancing act because unlike in the UK, it just about manages to keep the large chains within the general IPU body. Whereas in the UK really the chains almost, you know there is a pharmaceutical services negotiating committee, and that's the place where all of them come together to talk about terms and conditions. But they don't talk within the National Pharmacist Association about what the chains want, they talk about what the independent members want. So you know that that sort of arrangement means that in every such a body inevitably would have to make compromises in in the position that it took. So the point of having a professional body is that there is one that simply talks about professional matters irrespective of what this means in terms of the conditions under which pharmacists necessarily work or whatever on the one hand. That it is actually outside to all intents, political discussions. So when a professional body doesn't necessarily talk about going on strike, whereas obviously the IPU felt some years ago that it had to go down that road and say we will not continue to fulfil the terms of the contract, because the other party is not fulfilling their side of the bargain. So it is very difficult to be able to square those circles. It is fine to say we would have, I don't know an alliance, in which all of these bodies would come together and say, OK, you know, this is something that we think, and there are examples of that in the United States, which you know, it is obviously not really a country, it's a federal group. So you have every individual state, you then have a set of national bodies and all of those bodies are separate, well they have some alliances like that which bring together all of these groups. And they every now and again come up with something and issue statements and make documents and so on and so forth. So I think that you have to accept that. You know you either have a professional body which only does professional things, or you have something which is part professional body part something else.” **(Participant 10)*** *“I think just merging them and taking what we have can be dangerous, because you can miss something you can over represent from one area. So I think I think we need to use certainly all the excellent bodies that are there, but in a way that it's new and it has a new direction and new definition of what it is and if it follows suit then that the people who are needed and the roles are needed can be filled absolutely.” **(Participant 11)*** *“OK, so my sense is that, you'd have to talk to the stakeholders, because I think if I was going to go about it, that's what I would do is we'd have to take soundings because I don't really know what would be the best model. I have only ever had positive interactions with the IIOP. I've had very little interaction with any of the other professional bodies like HPAI or the Irish Medicine Safety Network or PIER. So I don't really know, or the IPU again, I've had positive stuff. But what you don't want I guess, is that you know if they all go together that there's this sense, that, oh well you know due to the representation, almost proportional representation of community pharmacists in just by virtue of the fact that they're probably the largest group that they get to run the show. And that everybody feels like was just the IPU with another name on it, do you know what I mean?” **(Participant 5)*** But I do think I think that there's a lot of the people who advocate for a professional body. And it's often the case that people clamour for an organization to be established to do something and I don't just mean in pharmacy, but if you listen to the news, you get protesters saying we need a new commission on this and an agency for that, or an authority for something else. But they actually want something done. The clamour isn't always to set up an organization, it's to get something done and they think the first step has to be setting up an organization, but you can do things with the coalition you can bring together coalition of existing players and existing stakeholders and get things done. All you need is a shared agenda and a determination to succeed. **(Participant 4)** *“No I wouldn't have the IPU or the HPAI have any hand, act or part in the formation of this new body. They could maybe sign a memorandum of understanding to try and work together at times but I would not give them any officer roles, I would not have them on the boards, I would have been completely separate.” **(Participant 7)*** **(4.)**Use of the PSI - “I would strongly feel we do need a professional body to advocate for all pharmacy, not just Community, Hospital, industry, it's a professional body that advocates. And the GPhC and the Royal Pharmaceutical Society in the UK have done that, they separated….I actually think it could be done without separation by the PSI, if the PSI broadened its remit…I know, the PSI for example sees its remit as protecting patient safety, but I think that remit needs to be broadened to also include advocating to what the role of the pharmacist is, and what the pharmacist can bring as part of a multidisciplinary team. I personally think that it is a multifaceted role and that it's more than just patient safety.….Now, it is interesting when you look at other professional bodies and I look at the Irish Nurses and Midwifery Board and they have managed to have the professional lobbying and the regulatory under the…same organization. So I do think that it can be done if the structures are done right……..And again, if you look even to Australia, it is the professional arm of the profession, the professional representative arm of the profession that lobbied for those, and then the regulator with them brought in the criteria and what was required to achieve that.” **(Participant 3)** “I think there could be two arms of the PSI, one to do with advocacy and one to do with regulation.” (**Participant 3).** *“No, I would start carte blanche, I would create a new body similar to what they did with the RPSGB, many years ago, and there was a divergent there, and it's worked quite well. So UK pharmacy tends to be 10–15 years ahead of us in everything. I think part of that is because we don't actually have a body to keep us in line with the modern world, and so I would actually just copy what they did in the UK because it is a template that has worked and you start to see things coming from that like say the GP pharmacist that we could totally be doing here now like straight away, or if there isn't actually anyone to positively lead that, even though to be fair there are some GP practices now, ones and twos starting to recruit pharmacist because they see that the idea works in UK, that the science is good, that it works and it's mutually beneficial thing.” **(Participant 7).*** *“But I think we could draw from the knowledge of medicine or dentistry, your nursing or other professions, who have good well-built organizations and get people from those organizations to come in and help us get up and running and get the fundamentals right and they might just be on the board to help you know to support, the initial structure. And you know, if we look around there will be people in this country, who did this job in other countries, you know that kind of way……New Zealand, you know or Canada and supporting our organization sitting in on board calls that way.” **(Participant 9)*** *“I think we need professional representation and I think the only way we can do that is for the profession to come together and decide how they want that to be manifest. A professional body is one way of doing it, is it the only way? Maybe it doesn't have to be a body. Maybe it's a collective forum? Maybe it's appointed or elected committee? Really it would depend on what you want that professional bodies to do. So I agree, professional representation agenda is required. How it's done, I wouldn't really have any affinity to one model over another provided that the principles would be employed such as, there is elected representation, the profession represents them, the profession drives it, the profession owns it, the profession pays for it, the profession commits to it, and the profession carries through on it. So my worry would be an organization deciding to set up a professional body and it not being driven by the profession” **(Participant 1)*** *“If you think about the medical model. You know I see GP education training strategy, career development, workforce development that lives there [professional body]. And then they have, you know the IMO, who are their union, and they have the GMC who are their regulator. And I think, it's really clear that there is a space for different perspectives, and actually the strength is that there are the three perspectives. For any of the colleges, the medical colleges they are responsible for the education, they set the curriculum. Now and then the regulator takes that expertise and I should still be registered against those criteria and the policing of that possibly needs to be done, or rather I need to go in with my badge that say I'm eligible to come to be regulated and to be registered within the profession, because I've completed these criteria, but I think that model is well developed for the other professions, and I've not seen a reason for us not to go down the same way.” **(Participant 8)*** *“Well, the point is that the Pharmaceutical Society of Ireland answers really to the Department of Health. So really and truthfully, why should the Department of Health be deciding whether and what sort of professional body pharmacy has. That's for pharmacist to decide if they want to professional body, and what form it should take and what roles it should exercise. And whilst the arm's length thing kind of works. It's only at arm's length, there is still input. I mean the PSI goes to the Department of Health and tells them what they're doing. So this is what we plan to do. This is our business plan for the next five years or whatever. And this is how we see this. And if somebody in the department I am not saying that they do, or that they would, or that they would have any ill intention. But if somebody in the department says, well, look, you know, I mean, why on Earth are you doing that? Why are you not doing something like this? It is simply that it's not appropriate…if the professional bodies is to advocate on behalf of the profession, it should begin within the profession and be for the profession and about the profession.” **(Participant 10)*** *And it's up to it to survive, it shouldn't be part of and it in essence dependent upon permission from, however easily that permission is given from either the PSI or the PSI's ultimate director, which is the Department of Health. Don't forget that that the IIOP also has to work with the Department of Health and with the HSE I mean you know the education for vaccination is excellent, but it's done as a collaborative effort, and you know the IIOP has to go to the department essentially with the PSI and say, OK, you know, what do you want us to do. You know the HSE comes along and says, you know, we're going to be doing these things. These are the department and the nationally approved services and programs that will be on offer and you know pharmacists are supposed to be doing this. That's very different from pharmacists saying ladies and gentlemen, this is what we can do. **(Participant 10)*** *“We don't need anyone's permission to start a professional leadership body that unites the whole gamut of Pharmacy. We only need to self-assemble**.” (Participant 12)*** *“Well I don't know, I suspect Department of Health are in there somewhere like it was Mary Harney, who changed the PSI to its current configuration. Is it that the Minister for Health, would say actually pharmacy needed professional body and make it happen? Because in some ways, again go back to it, your DoH is your strategic authority, but I don't know that level of governance, you know what I mean, I know it to here, but above again. So who are the decision makers and who has the authority? Because previously the PSI was founded, it was Mary Harney and Ambrose is my understanding. So I suppose my question would be and also you know if the HSE come back saying our strategic workforce plan has a gap like we don't have enough pharmacist coming through.” **(Participant 8)*** *“Well, it's possible that a professional leadership and representative body could have been established at that time, but I would argue it wasn't the role of the Department of Health or the Government when they were passing the legislation to establish it. Because professional bodies like that don't normally have a statutory basis, Britain is different because they've got a royal family so they can stick royal in front of anything and then it sounds official.” **(Participant 4)*** *“I personally wouldn't be advocating that we go back to the drawing board. I think we could look at Canada, we could look at, the UK, we could look at Australia, what has worked for those authorities in those jurisdictions. We're not a million miles away from what they are doing, I think we could see what has worked well and implement it here. And there is talk and I think having a consortia for a professional body that is bought into by the three Schools of Pharmacy simultaneously, having something that is collaborative and you need. If you were establishing a professional body, you need the key stakeholders on its steering group. You need the Schools of Pharmacy, you need the IIOP, sorry the IPU, you need the HPAI, you need representatives from those to be, to have I suppose buy in and people invested in it. But I personally feel it doesn't have to be a million miles away from the PSI.” **(Participant 3)*** *“Yeah, I'm very familiar with the RPS and with the work that it does, as I say it does high quality work, but its membership is not growing. If anything it's going backwards, it has struggled financially for years, even though it's like an incredibly wealthy organization, but it's run at a deficit for years because pharmacists aren't universally excited about the idea of funding it, and huge number of the pharmacists that I know in the UK feel that it is divorced from their interests. You end up then potentially kind of ivory tower type organization, which is advocating for things. So as I said at the outset, the RPS does some great work, but it isn't seen as belonging to all pharmacists and it can be seen as being a bit academic and a bit theoretical. So there is a role for it there, but I'm not wildly enthusiastic.” **(Participant 4)*** *“So until, what I think should happen is there should be a coalition of the willing of willing pharmacists gathered and whether that's from academia or regulatory, or IIOP or wherever this coalition of the willing, much like the blueprint for pharmacy in Canada, where there was all stakeholders around the table saying these are all the jobs that need to be done, these are all the people who need to do their particular jobs, who's going to do the professional representation, who's going to do this? Who's going to do that? And there needs to be the establishment of a professional body.” **(Participant 1)*** *“I would yes. I would be in favour of the development of a national Irish professional body for pharmacy. I guess I would look to bodies…….the likes of the Royal Pharmaceutical Society in the UK. And I would absolutely see….and that is not in any way to take from the work of our own Irish Institute of Pharmacy, which I think do absolutely stellar work. I think that it would be helpful to have a body that is resourced beyond what the IIOP is currently resourced and that would have a broader terms of reference, you might call it or you know remit which would provide you know greater support than is already there in terms of professional development. And aligned with that could be something around professional credentialing.”**(Participant 13)*** *“They can all sit within one single organization, or they could have different roles, housed in multiple organizations, but there needs to be…..you know, the blueprint for pharmacy in Canada would be a very good example of how they use the collective dispersed in different areas for collective gain.” **(Participant 1)*** *“Yeah, I think the there are some differences between ourselves and the UK. Remember that when the RPS was under formation in the UK. It had assets, those assets came from the original RPSGB, so there was both support and resources available, as well as a period of time for the RPS to be created, and in that period of time, all of the different pharmacy bodies in the UK were consulted, they took part in discussions, they decided what they were going to do, and to some degree, what came out of it was therefore a body which most of the existing organizations agreed and felt offered some progress. Under those circumstances it was feasible for the RPS to carry out this development function in terms of education. However, it wasn't absolutely necessary that they did it, they could have decided to do it differently, they could have decided to have an educational body, which administered the process of CPD separate from one that set the standards for CPD. Which remember in the UK are not actually just standards for CPD, they are also attached to a framework for the development of pharmacists. And unlike here, that framework Is recognized by the National Health Service. So if somebody says they're an advanced practitioner, that the National Health Service recognizes that fact. And people going on, you know the role of a consultant pharmacist, is not only a role that the RPS recognizes, but it's one which also has a job description within the NHS. So all of that is different in this case, there was what the funding……the resources which are available for the IIOP come in part from the PSI. Right? So if IIOP separate from the PSI, do those resources go with it. How do they go with it? So there are a variety of things there which make that whole process a lot more complex and difficult.” **(Participant 10)*** *A]s I say, I think the key aspect is what roles do people imagine this professional body taking on and to what extent are they prepared to see a professional body being distinguished from a regulator or another type of representative body such as the IPU or the HPAI. And I think there's a certain confusion about that, and I think with the IIOP essentially was really, it partly comes out of the PSI's need to develop the profession as well as the other side of it, which is to ensure that the pharmacists who are practicing are fit to practice. So it's a combination of those two things. The reason for it being at arm's length is because if it's going to help develop the professional, and there's going to be some standards for that, then they needed to be able to essentially outsource that. But I mean originally the intention for the IIOP was that the IIOP would fulfil a sort of professional body role, because they intended it to have the power to designate individuals as fellows and various other things, which would be typically what you would see in a professional body. So you know, I'm not surprised that there is a degree of uncertainty and confusion and I also think that when you know people look at the UK, they imagined that it is much more similar to us than in fact it is and that doesn't help them. And of course, obviously we've got quite a lot of pharmacist in Ireland who graduated in the UK, and many of whom are members of the RPS, I imagine and therefore look to it and say, well, you know, I want something the same here. But I'm not sure that you could have something quite the same.” **(Participant 10)*** *“Well there are two things there. One is you have to have somebody who leads in terms of talks about the matters that are important and essentially leads in the discussion of those things. And then represents the Members views to whoever else they have to talk to. But then there is also simply the running of the organization, the making sure that the operations work. Well those operations side of it does not necessarily have to be done by a pharmacist right, however the leadership the ultimate leader, president, whatever they're called of such a body, yes, they do have to be because they should come from the membership. And even if you look at, if you look at, say, the Irish College of General Practitioners or some of the other medical bodies, you find in actual fact they have General practitioners are the president. But the CEO is not necessarily.” **(Participant 10)*** *“I absolutely think pharmacists form an integral part to it, but I think yes, to answer your question with a yes or no sort of binary…does it need a pharmacist? Yes, absolutely and I think a panel of pharmacists over different experiences coming from academia, community, hospital, industry, but I think it's really, really important that we have collaboration with our peers, you know from a medical point of view, you know social work, dietetics and all the MDT, because I think we work in teams and I think it's really important we have a sense of that. So whether it would be you know on a consultation basis or you know there are exact role would need to be fleshed out. But I think it's really important that we don't operate in a silo.” **(Participant 11)*** *Absolutely, you're not a profession, if we're not led by a pharmacist and by the way you know yourself now you're in the only school of pharmacy that has a professional pharmacist leading the school. Trinity is now, it's not even called pharmacy anymore. We're pharmacy and pharmaceutical sciences and the college of surgeons is pharmacy and biomedical sciences. So we are an “and” for profession.”**(Participant 12)*** *“Oh yeah, I think the leader needs to be somebody of a high national standing and strong I mean that's the key point, they need somebody to be able to bring all these groups together. So it to me yes it needs to be a pharmacist to represent. My expectation is that they would be a person to if the government are looking for advice on pharmacy related or pharmaceutical knowledge related to drug usage and in the community, that's the person that they should be reporting to. And this is why and we've seen this from other organizations and in medicine, this is what they're doing. But in terms of an expert on drug use and medicine use, this is what and if the government needs that, that's what we're doing and that's what we provide I would have thought. Now the HPRA provide that, but they provide on what medicines are licensed, but I would have thought and there is a role for that. So, that's what I would say on that regard.” **(Participant 2)*** *“Oh, just in question, intuitively I would have said it has to be a pharmacist because I think they would have………well they don't have to defend stuff. I mean, I think immediately if you're not a pharmacist, you are on the back foot a little bit. You're like, well, sorry you're the voice of the profession, you are not one of us, so what's that all about? So I, I think it would really be important to have somebody who is a pharmacist, yeah, but not only that. You know, I don't think that's going to do it, I mean, you'd have to have a really charismatic, really amazing person to do it, because, again, if they're the flagship, they'd have to be able to negotiate with pretty much you know all of the other groups and bring them into the tent and reach across the aisle and every other metaphor you could think of, you know.” **(Participant 5)***
	Functions & Policies	*“For example, one of the areas that we don't have yet in Ireland is pharmacist prescribing and I think while that needs to be regulated, I also think you need a professional body that can develop it and develop the competencies around it and have oversight of the skill set, set standards, all of that, which it's not the role of the regulator to do that. It is the role of the regulator to make sure your licensed, to make sure you are competent, in the sense that to actually check on those things and make sure that the appropriate people are doing it. But in terms of actually setting standards and developing a skill set, I think that's where the role of the leadership body comes in, separate. I mean, they will always work together in that sense, but I think they have to be, they have to be separate entities**.” (Participant 11)*** *“And the IIOP CPD isn't actually, doesn't allow credit accumulation, doesn't allow micro-credentialing, all these things need to be looked at in an in a new professional body and a new professional body then could award membership or fellowship of the profession. When I qualified 20 odd years ago, you were able to do that in the UK. You were able to accumulate your training and apply for membership of the College of Pharmacy Practice. And that's gives somebody that gives somebody an aspiration and something to achieve to.” **(Participant 3)*** *[T]he professional body provides structured training… guidelines…supporting structured training placements. Facilitating the profession to support this which is giving back the profession.” **(Participant 2)*** *“It could fit into a professional body role, it doesn't have to. I mean there is probably just about enough separation between the PSI and the IIOP to enable the IIOP to function. It's not an ideal circumstance, but then, it wouldn't be necessary if the IIOP were separate from the PSI for it to actually be associated with the professional body. It could exist as a separate body itself. So there are pros and cons to it being where it is, there are pros and cons to it being attached to a professional body and there are also pros and cons to it being an independent body in its own right. I think the professional body, whilst it would have views about continuing professional development and so on, doesn't necessarily need to administer that, or doesn't necessarily need to take on that function in order to be able to express those views and to influence what happens.” **(Participant 10)*** *“I think what has happened unfortunately is, and we're all guilty of it….I think the Schools of Pharmacy see it as an opportunity for themselves to get some power through Appel, they might do it that way. The hospital pharmacists association don't want it because they feel it will threaten their power as a union. The IPU are the same and everybody is doing stuff they shouldn't be doing……..the Hospital Pharmacist Association set up its own educational training wing……we establish an annual conference and that became our source of income…. there are special interest groups in psychiatry and total parenteral nutrition, aseptic…..But unfortunately all of those things belong in a professional body, not in a union right. Now it doesn't really matter as long as all it is a sort of an informal special interest group, but when the rubber meets the road then you're in trouble. And there's a really good case study of this as an example, which is these standards for aseptic pharmacy in Ireland, right.” **(Participant 12)*** *“additional services being credentialing, career structures, coaching, involvement, education, whatever, fellowship**.” (Participant 1)*** *“As I say, to represent pharmacists and to talk about pharmacy as a profession, you don't necessarily have to administer the process by which they update their skills and maintain their professional development. You could in actual fact, the professional body could simply be the body that sets out the standards and the framework for that process, but for it to happen in Ireland, that body has got to have resource is coming from somewhere.” **(Participant 10)*** *“Well, once again, it depends upon the types of roles that it takes on, but if you're talking about advocacy right, which goes with representation, then advocacy usually involves talking and writing and presenting the views of the group to other people. And there's no reason why you shouldn't have such a section right, and why they should not present on behalf of industrial pharmacists or whatever they're called, from within that professional body. And equally, there's no reason why they're the head of that professional body, the President or whatever they're called cannot therefore say, you know, our members who are industrial pharmacist or academic pharmacists or administrative pharmacist, whatever they are…..well, uh, you know it would only be right that they that…..” **(Participant 10)*** *“Well, I think in terms of CPD it does an excellent job. So I think it [IIOP] does an excellent job and I mean I, I think within the constraints and under which it must work, it does have a really very very good job. But as they say, it was supposed to have other functions. I mean it was supposed to be involved, for example in what you're doing in research. Right? But ultimately the PSI it seems must have said well, we don't have the resources for that you know and you should just get on with the education side of it. And when the PSI does research it usually has a fairly clear idea of what the findings of the research are going to be before it actually gets to the end of the research.” **(Participant 10)*** *“I think you know there's certain aspects that we really need to focus on, like prescribing that we've been left behind on as a profession. And I think it's important that we really use something like professional body to you know, to gain traction on areas of practice that are kind of under representative currently.” **(Participant 11)*** *“Absolutely 100% yes. I think there's a lot of areas for, I suppose I've worked in hospital pharmacy for nearly 20 years. We don't, you know, have a career pathway at the minute. In fact, it's about to go to ballot unfortunately for strike action in the whole area, and I think that's where a lot of frustrations lie that you have people who have developed a lot….focused a lot of practice, one particular area, but don't have the recognition for that speciality. And they've also spent many years developing their skills in a particular area, but there's no developed frameworks, there's no assigned competencies, and if you look I suppose I look in my area to the UK and Australia and Canada, to see that they have a lot of standards in place and even like whole time equivalent, you know recommendations all those kind of parameters that we need to set for specialty.” **(Participant 11)*** *“So I think yeah, I think yeah career structure and development. And I can only say this from a hospital pharmacy, because that's where my all my experience is. But I would think you know career credentialing, and I think maintaining some form of CPD is fundamental to what they do. Be that portfolio review, peer-peer practice etc…..built on that. But I think you know practice frameworks, like the NHS have for example clinical leadership competency frameworks for people in leadership roles. I think similarly, if you're in different areas of pharmacy you need to develop practice guidelines frameworks, competencies and also the other skill sets that aren't directly clinical. So for example, leadership, mentoring, coaching all those avenues, and I think the IIOP have put a huge body of work in already. The other thing is practice standard, so what is it? Like how many pharmacists whole time equivalents are needed to run particular services. I think we need to develop KPI's around practice and I think a leadership body could really get involved in that and develop those aspects.” **(Participant 11)*** *“It would be convening committees that are positioned to provide the profession's stance on clinical issues. It would then take those stances and feed them into the appropriate areas of the healthcare system. It would partner with the healthcare system to identify the evidence that supports the progression of the proposed systems. It would help with the collection of evidence. It would upskill people for those roles and it would support advanced practice within the pharmacy population. There would be career structures that would be extensions of the existing CPD system.” **(Participant 1)*** *“Well the roles and responsibilities are to represent the members, to speak out on behalf of the profession, it's different sectors, community, hospital, industry, academic and so on. And to speak therefore on behalf of pharmacy as a profession. What it can do, what it should be able to do in the future. How we could develop and how it can contribute.” **(Participant 10)*** *“[A]dvanced practice specialization, advanced generalists, and that could all be supported by a professional body. And I think it's better placed there than in the universities, because I think, with the greatest of respect to our academic institutes, the delivery of education in service of the education regulations, and in service of HEA requirements and in service of university metrics is extremely different to delivery of education in service of a professional agenda.” **(Participant 1)*** *“A profession needs an organ to disseminate views. Prior to 2007 there was an Irish Pharmacy Journal, operated by Kenlis Publications, a registered company owned by the PSI. The loss of the Journal was a great disappointment to me personally, as I had done considerable work to raise its profile and to make it financially viable……….At the time of the reforms, I was assured by the then Registrar of the PSI that while Kenlis would go, the Irish Pharmacy Journal would continue under the auspices of the PSI. However, it soon went to PDF only with no hard copy distribution, and became defunct in 2010**.” (Participant 12)*** *“IIOP kind of, they don't do a huge amount of like CPD itself, they tend to do more about how you do your e-portfolio review and then they do a little bit. They've gone down a tangent around wellness and self-help because a lot of people in the profession are unhappy in the profession. So they're doing a little bit on that, and at a lot of that is driven from this void that they have nowhere to go. They've no, support, they feel like they have no anchor, they feel isolated.” **(Participant 9)*** *“So I think if there was an organization like this, possibly, maybe it might go to some degree to help people because then least they have somewhere to go, and you could bring the benevolent fund into it…..But like most people wouldn't know about the benevolent fund, and the work that they do. It was part of the old PSI, when the PSI was more of a representative body and then they became the regulator and the Benevolent Fund is still kind of there, but it doesn't sit within a regulator. It sits within a representative body. And the Benevolent Fund will be there to help support people, in lots of ways, so you know you could bring them in and maybe expand their role to support people in more than just financial crisis, but with any other crisis that would be happening in their lives.” **(Participant 9)*** *It is common practice for professional leadership bodies to award Honorary Fellowships. Fellowships are one of the artefacts of a profession, part of a culture of professionalism. These awards are prestigious and bestowed upon individuals to honour their outstanding contribution to a specific field, or sometimes more generally to society. One of the things we lost with the 2007 Pharmacy Act, was the ability to nominate peers to Fellowship….. In the 15 years since then, there has been no opportunity to acknowledge pharmacists who have made an outstanding contribution. Meanwhile, such recognition continues to be bestowed in the medical profession, for example.” **(Participant 12)*** *And so I think the idea of you know regulation and registration probably you know needs to remain with the PSI, but I guess what I mean by credentialing and would be something along the lines of what was introduced by the Royal Pharmaceutical Society a decade ago. And you know, when they introduced fellowships, for example. So memberships and fellowships. And something that would be indicative, you know that would provide, you know, postnominals, I guess for pharmacists who achieve more granular expertise within a given area or you know where it has been demonstrated that that they have reached a certain level of mastery, or are competent to perform within a particular sector or a particular area of work. And you know that that could be done on degree of gradation. So as I said, I suppose you know the PSI are responsible for entry onto the register, and retention, you know for the governance of retention on the register. But there is no segregation I guess within that register you know it's dichotomous, your registered or you're not. And where I guess what I would see in terms of credentialing would be something more along the lines of the professional membership in terms of the likes of a Royal College you know, or whatever synonym you want to use for that. But that would be indicative to you know, lay people and interprofessional colleagues, you know of a person's attainment within a particular area.” **(Participant 13)*** *So yeah, I guess I think, education most definitely would be a role, and credentialing, you know. And again I would, sort of look to the model of the RPS for that you know. So I guess at the moment you know the IIOP have their role in in terms of the administration of everything you know with regard to the e-portfolio, and then you know the administration of the OSCES or whatever for people to remain on the register. And but I think again that comes back to the dichotomous on the register or not on the register, whereas what we're talking about here a more granular credentialing of experience or of specialization.” **(Participant 13)*** *“I think there is opportunity for professional advocacy……that happens to a degree at the moment you know, through the IIOP to some extent, and but again limited by some of the constraints that are in place and it happens to some extent through the unions. But the fact that it happens through the unions, reduces the credibility of those arguments, I would say, you know, at policy making level and at government level. And the fact that it happens through those unions also dilutes the integrity of the need for that extension of professional scope of practice, because the primary variable of discussion is the money and you know and forefront you know issue is around the remuneration. And I think that that disables you know the conversations that could otherwise be had. That's not to say that the economics of it is isn't an important issue, it is, but I think if we were able to make the arguments in the first instance, with for example at the integrity of supporting patient safety as the primary focus, you know. Or the government getting return on investment for their funding that goes into the HEIs, you know to support the development of these pharmacists. If we were able to, you know come at that argument from a different angle that doesn't look like you know we're just we're just looking to, you know, get on the gravy train. If we were actually able to rationalize that out in a way that says, listen, you know, let's look at this from a health economics point of view, you know, but let's take consideration of what it is that that the profession of pharmacy as we train them under the remit of the EU legislation, let's take a more holistic look, you know at whether we actually could exploit that resource, you know better than we currently do**.**” **(Participant 13)*** *“[I]t's [professional body] role is about ensuring that pharmacists that their guides and guidelines that are applied” **(Participant 2)*** *“The main role, I suppose it's to advocate, it's to lobby, I suppose it is to advocate for the expansion of the role and to advocate, for, I suppose, the education and the training of the role and to also set the standards” **(Participant 3)*** *“A professional….they should be involved in the setting of those standards and involved in the setting of the educational expectations and also where the future professional will go. I think one of the key things they should be doing as a professional body is lobbying to have representation of pharmacist in the Department of Health, we don't have a chief pharmacist and it's quite a number of years since we've had a chief pharmacist in the Department of Health. All other professionals are represented there between nurses, dentists, doctors. We have quite a number of pharmacists working in the department at very senior roles, but they're not there to advocate for the profession or to promote the professional roles and responsibilities. I do think that they have, I think that they have a very strategic steering remit, so that they would draft, I suppose, future vision documents of where the profession should be aiming be at in medicines reconciliation on a formal basis, it might be for advocating for GP role pharmacists, it may be advocating for prescribing roles, prescribing authority. There's nobody advocating for these roles.” **(Participant 3)*** *“Yeah, well, as I said, it's in my view it's not so much an organization as a forum, and its role would be to bring together the leadership bodies across the different channels or the different career options for pharmacist, so the I'd be looking at PIER, I'd be looking at the hospital pharmacists, looking at the IPU for community pharmacists and coming together and say well what have we got in common, what have we got that is different, what are our common objectives? How can we grow our profession in relevance in professional scope? How can we maybe look at combining some of our CE and CPD efforts. For example the IPU hosts and runs the IPU Academy, which is Ireland's largest provider of continuing education. For pharmacists we have Members in the IPU Academy who aren't community pharmacists. You know because we allow hospital pharmacists and others to join it, but there's potentially more that could be done, if we had the full width of the profession involved in that. So that's just that's one example, and it could be that we could agree joint shared policy on medicines reconciliation and care of particularly transitions of care community to hospital, hospital out to nursing homes. Nursing homes back out to the community. There's a role in there for hospital pharmacists and community pharmacists, and potentially medicine safety pharmacists from the HSE to come together, draw up a coherent policy, a coherent policy position that supports patients, provides greater safety and actually advocates for what pharmacists can do in that space. Those are just examples.” **(Participant 4)*** *“I suppose what I would like is I would like a voice for pharmacy, I would like to see somebody who is you know, as head of a professional body that is I suppose interacting with you know the Department of Health on behalf of pharmacists. Who's lobbying on behalf of all pharmacists to move our profession forward, to bring us into, I suppose, mainstream. And it it's not just about PR, but I would think PR is a big part of it. You know, telling people what to pharmacists do, having a public persona so that people say, oh, that's man or that's that woman from the professional body of pharmacy they do great stuff, I didn't know, did you know pharmacist did this or you know I really feel that PR piece is missing and I think that a professional body should be definitely be a positive representation of what the profession is all about. I think it should be responsible or certainly in tune with what is best practice, you know, in in terms of looking across at other jurisdictions, what's happening, you know, maybe in North America what's happening. In other words, what are pharmacists doing that we could be doing? Why aren't we doing it? Is it right for us? You know, given our culture, given our, is it the right fit? You know what I mean, and I'm thinking about the future of the profession, like it for an example………………so in terms of I was just saying about horizon scanning, so I think you know trying to improve pharmacy trying to, you know, see for pharmacy in Ireland that we could be the leaders in,” **(Participant 5)*** *“So I suppose the whole idea of professional standards and you know and supporting people to learn, develop. Supporting you know career development and role development and the provision of you know educational support would be things that I would see as being important.” **(Participant 6)*** *“Uh, I think the professional body would probably need to start looking at retention of pharmacists in the profession. I think increasingly there's a massive leakage out. I do a lot of preceptor for a lot of interns and had well over a dozen in last years and I really can't see and I'd say at least nine of them don't see any future longer term in pharmacy, because I think it's so limited in in ways and I think the professional body would really have to address the leakage out of the profession of pharmacist, and why are they going? And the reasons for that. And kind of looking at expanding things because I think like if there was opportunities like say GP pharmacists, maybe supplementary prescribing pharmacists as a starting point. I think that would really help their retention. And I think like say for example, if you have a nursing home and you were supplementary prescriber pharmacist, so you're in community, you are servicing your nursing home contract and you are supplementary prescriber, and you go into the nursing home and review round maybe once a month or whatever is appropriate. I think like for the pharmacist delivering that ward round, they would get a massive sense of kind enjoyment and benefit from as well as well as. Obviously the patients and the clinical teams benefit from it as well, but I actually think something like that would basically retain people in the job longer, because it's kind of proper use of skills.” **(Participant 7)*** *“System is working in pursuit or should be under their professional duty of care working in pursuit of only one objective, which is the enhancement of patient care in the use of medicines, minimizing the harm and maximizing benefits of medicines usage.” **(Participant 1)*** *“So for example the IIOP was as far as I know it, it responds to a PSI need to recognize we need that, and that that that need should have, they didn't go the whole way, they didn't create a professional body to do that, they just created a funding mechanism to fund the IIOP to provide CPD, which should have been captured. So the tasks of the IIOP should fit under a professional body, but we've already gone to, there's too much water under bridge to try and rectify that and it works, it's fine, it's functional, so my feeling is we can't change that and is there any point? No, I would say not.” **(Participant 2)*** *“But it [professional leadership body] has to also engage with the structures of the day that are outside of it. So if the structure is at the moment are the Department of Health and the HSE primarily right. Then you know…so the IPU have traditionally of course engaged with the department health on the contract, right, but the contract isn't everything, and there's the professional side of things, and we know as well that like we are not adequately remunerating community pharmacists for cognitive services. We don't reward people….we have no structures for doing medicines reviews for example, or any of those kinds of things and so, like and. And we're not very inventive, or, you know, I haven't seen too many pilots of those kinds of things going on. And in a way, like you know, there's a pilot going on at the moment. The iSIMPATHY thing, which is in Northern Ireland and Scotland and here, and the HSE are involved in that. That's the medicine review project**.” (Participant 12)*** *“But what we don't have, what we're missing is a professional body professional representation, but in my view there is no point in having a professional body if there is nobody to whom that professional body can speak, or nobody with whom they can negotiate, and so even if the IIOP decided in the morning everybody is going put their hand in the pocket, we're going to get a way in which to be able to represent ourselves professionally. We're speaking into a vacuum because there are no counterparts in the Department of Health or in the HSE, so it's nobody's job to talk to a professional body” **(Participant 1)*** *But even if we were all together, really strong, really collaborative, really professional, we then have a big job to influence the system to be ready to receive the pharmacy input, because at the moment it's nobody's job to do it and my experience is that if it's nobody's job to do at, nobody will do it or put themselves out. So even though if somebody may absolutely understand the need to have pharmacists, if it's not their job to do it, they'll deliver on their own key performance indicators, and there isn't any incentive for them in the public service to put a foot beyond that, because all they bring is a world of pain to the detriment of their own job. So if there isn't a counterpart at a national level to engage with a professional body, it isn't going to go anywhere.” **(Participant 1)*** *“Yeah, I think one thing we should be looking to do would be to do is to map the connections between a professional body into the HSE and to the department of health, you know. And that's another big vacuum that we've got is the Department of Health has no chief pharmacist for years now, and I think that's been a huge miss as well, because we just don't have anywhere to engage and if we had a professional body, it might help to alleviate some of that.” **(Participant 12**)* *“But I guess what I don't know is because we don't have that DoH pharmacist who makes the decision and who says there's a business need and who says that actually the health system requires more pharmacists come out of our universities and then goes out of the other way around, you know?” **(Participant 8)*** *“The strategic decision maker for the HSE, it's not….. actually I wouldn't agree that it's a Chief Pharmacist in the HSE. Strategic decision making in the Irish health system is Department of Health and within Department of Health there is a perception that the role of the chief pharmacist was legislative and not practice and that's what I mean, there's a view that is very narrow about what we do and they don't get it. And I think that until we have a chief pharmacist within Department of Health we have a gap. Because once you have a chief pharmacist within the Department of Health and strategic policy, the HSE operationalizes Department of Health Policy and until there is a policy that comes through and says you need to have pharmacists in this way and you need to operationalize these things, and you need to have them in your models of care and you need to have them in your clinical programs, and we expect to see this back. It is an unknown unknown to HSE. Individual pockets where they know pharmacy, they have pharmacy and they value pharmacy and they defend us and they won't allow anybody take us, you know….they just absolutely this is what our pharmacist does and know that's what they do and go away…this is, you know, we want our pharmacy service. But then you have the places who really haven't had access to a good pharmacist or any pharmacist and they actually don't know what we did. They think we dispense tablets, and they're in an office, so why would I get one of those people in the door here.” **(Participant 8)**.* *“It really depends upon what people see the….what the feeling is about the functions and the roles that this professional body is going to take on, right because if it's going to a degree, that also depends upon what recognition it has from the outset from the Department of Health, HSE, and so on, right. And unless it has some degree of recognition from them, it's hard to see how it would therefore be able to fulfil some of those functions.” **(Participant 10).*** *“Now everybody will tell you we need a chief pharmacist. A chief pharmacist is not going to be able to do it…..We need pharmacists embedded at the model of care development stage, because once those models of care is established from the national clinical programs, that dictates what's going to happen in that clinical agenda for the next 5, 10, 15 years and there's no going back to rewrite the blueprint. All the pharmacists that are brought in at that point are just purely implementing.” **(Participant 1)*** *They're simply a group of professional that they need that connections at the higher level to be able to influence that and this is an important component of the role of a professional body that a chief pharmacist representing those is on, is at the decision making table, be that in COVID crisis and whatever. And we see that other organizations, as far as I'm aware, the IMO and so on and so forth, they have these kind of professional representation at that level.” **(Participant 2)*** *“And but I think as well, the lack of a chief pharmacist for Ireland in the HSE, same as there is a chief nurse, a chief medical officer, there should be chief pharmacist in the HSE to tie in with representative professional body as well. We are probably missing 2 pieces of strategic leadership.” **(Participant 7)*** *“Even for example, if within my own role, whether it's speaking to the HSE, for example, I have no representative body to support me to look for placements for pharmacy students, to develop opportunities for pharmacists or young pharmacists coming forward, because I have no body to give me guidance. So a single voice such as mine would not have the same power authority as a louder voice. So as you have with the nurses and you have with the medics. If they were going to the HSE saying we need placements, I'm sure that would have lot more traction, then me going to the HSE saying I need placements. So there's a huge void in in every area, so yeah.” **(Participant 9)*** *“One, the leadership capabilities because it will be a complex navigation; two, the professions appetite to invest their time, effort and expertise in shaping the health service; and three, a receptiveness from the health service and the DoH to engage with such a body” **(Participant 1)*** *“It's you know, the RPS, we -looked at it, has very strong connections in Westminster, very strong connections across the Health Education UK, very strong. It, you know, it has multiple, multiple, multiple stakeholders and most importantly if you were to write one thing in your thesis, the activities of the RPS are very much dictated by the strategy that's developed by the chief pharmaceutical officers in each of the countries. And if they didn't have that (and they have it incredibly well articulated in Scotland and Wales. It's not so well articulated by Keith Ridge in England, but when you see NES [NHS Education for Scotland) and when you see the Welsh Assembly saying what they want, guided by the chief pharmacist or Chief pharmaceutical officer), then a professional body can kick into action and deliver on that. But when you don't have any leadership around pharmacy strategic agenda at a national level, when you've no national strategy for medicines optimization, then a professional body is going to be left floundering unless it has extremely good influencing abilities”. **(Participant 1)***

Theme	Subtheme	Full quotations
4. Finance & Membership	Finance	*“So the only people who can fund this are pharmacists because the health service, if it funds it, has its interest; and if it's the regulator, if it's the professional body or if it's the representative body and if it was the IIOP, it would ultimately have to serve a PSI agenda because that's who it is funded by and the DoH agenda” **(Participant 1)*** *“So it would be easy to set up a professional body right now and establish itself [IIOP] as that in one sense, but it wouldn't be a professional body in the true sense of the word until pharmacists are willing and understand the importance of putting their hands in their pocket to fund a collective professional agenda, they are going to not buy into the ambitions of such a body.” **(Participant 1)*** *“And at present the only place it seemed likely it's going to come from, because the state doesn't seem to be have any particular interests in these things, would be for members to contribute.” **(Participant 10).*** *“So if you think about the pharmacy journal, the PSI would pay a fee of about £27.00 a year, which was roughly speaking 20% of the membership fee at the time. Now if that capitation fee. If the society was now required to give up a similar type of computation fee, from the 7000 members, right? Every year and that would go into the professional body. You could have the basis for a professional body to begin to exist and to work itself. But you see at the moment the problem is nobody wants to start this, because nobody wants put their hands in their pockets.” **(Participant 12)*** *“So you have that danger here, if we go to a professional body, and so you need to come up with some, I think it would be a good thing to have, a tax on the pharmaceutical society regulator in a way that the pay they should have to pay £50 or whatever it is per year towards the education of and the professionalisation of the profession out of our fees you know. Rather than giving people the choice of being in a professional body….. I would have it as a capitation payment at the start of the year from the society…. Let's say that the government could agree to capitation fee if they want, and then if people are at liberty to contribute as well if they want after that, you know. Like I would say, there's no reason why your pharmacist shouldn't donate to their professional body as well if they wanted to. You'd have to have rules for that and make sure that people weren't buying influence.” **(Participant 12)*** *“Now by the way I've talked to the GPs like the ICGP, they set up separately about 20 or 30 years ago. When they set up they established a fee of €1000 per year, plus I think €400 for CPD on top. Now GPs have a better income than the average employee pharmacists, right so and they had the ability to charge those kind of fees to their practices, right. But they have very high uptake, it's very impressive, how many people will join it. The same thing with the royal college of anaesthesiologists of Ireland. They have a fee of €400* per annum *for their members, so they have a small membership, but a significant enough fee, and then where they get their income to support their professional activities, is the fee income is only about 30% of their income to big share of their income is they get it from a contract they do with the Health Service Executive to oversee and manage the training of anaesthetist. So they look after the six years of training anaesthetist have, they accredited it all, they evaluate the people, they bring them in and they look after the whole program and in return for that to get an annual fee. So those are the kind of models that are there for how a professional body might work, and I don't know whether anybody is going to do it with our people.” **(Participant 12)*** *“[T]he other income source would be, then would be postgraduate training, the approval and accreditation of postgraduate training and the provision of it in hospitals and community pharmacies. And people would get paid for that. So for example, I will be saying to the HSE. Just like the NHS in England, you should be paying the community pharmacies for training your trainees, not just letting them pay them, and you should be paying them.” **(Participant 12)*** *“I would think that there should be some form of statutory funding and you know that that would support it. I do think that it would need to come from within the profession and I think it would be a body where membership is optional. You know, it's not mandated so. You know the PSI serve, I guess as that body and so I suppose I would see it as coming from an within the profession. And I could see an argument being made for some statutory report, but I realize you know that that there that there needs to be a lot of consideration around what statutory function is it then serving.” **(Participant 13)*** *“I think that funding could also come from employers, you know, I think that. I think that there could be, you know, something made of….I guess the professional development integrity and you know, and the credentialing that it would make it, you know that commercially, for example, if a you know, be it retail, pharmacy or be at a hospital or whoever it might be, would be able, you know, to use the fact that our pharmacists are all you know, say whatever the IPS, if you want to call it at the Irish pharmaceutical body or society that they're all credentialed by them, you know. Or to be able to say, you know, we've got people at the junior credential at the main, you know, and that and that our personnel are you know, across this. I think there's opportunity for funding there.” **(Participant 13)*** *“I think it's something we should consider, I think it's something we should look at. But at the moment there is a significant funding given to the IIOP every year, that I think would fund a professional body. There's up to €600,000 given a year to the IIOP to run it. And I think we have to see are we achieving value for money in its current status, I think that needs to be reviewed**.**” **(Participant 3)*** *“[T]here is some funding it could be with the assistance and utilization of some funding from the DoH, but there would need to be a membership fee from the profession as well, and then the success or failure of that body will rest on three things.” **(Participant 1)*** *“I looked at the College of anaesthesiologists…..they were part of the Royal College of Surgeons at for a long time as the Faculty of Anaesthesiologists and they just wanted to move out on their own and create a new body. So they did that, I think about 15 years ago. So it's quite new and it worked for them….they established income streams for it…..often rented it out as a board room space…..They also built a training and Education Centre out the back of the place, which accommodates about 80 people…… But again, their biggest income stream is the deal with the HSE and in a way, that's a risk, you know. But on the other hand, the HSE need them, you know, and it's in the HSE's interest to keep them happy and to make the training program work, because it's the training program. You're thinking when you think about it. It's not just anaesthesia itself, but it's intensive care medicine too. So they're responsible for the intensive care training program and the anaesthesiology program, so they work with those two programs in the in the in the HSE program structure. And that's where they have that connection into the HSE where there's a direct kind of a* quid pro quo*, you know? So the HSE are paying them money, but they can see what they're getting in return. They're getting trained anaesthesiologists…. The College of Anaesthesiologists of Ireland started in 1998 after splitting from RCSI. They get half their annual income from HSE Anaesthesia Programme and ICU programme. They are the self-organised advanced training and professional practice body. They tell the Medical Council who to put on the Specialist Register for Anaesthesiologists.” **(Participant 12)*** *“I think we're not very big, and yet, we should be big enough that the fees we pay and that includes the pharmacy registration fees for premises should be supporting the development of the profession. So it's a little bit like having you know the money in the bank should also be about the future and not about the current. Now that would mean my fees would go up, I imagine, I can't see the PSI dropping. But then again, some of the education stuff would come out from there and go somewhere else. So I would say I can't see us being big enough to sustain two independent funding structures I think it would have to be an integrated thing. I think you would go off and you would scope where the anaesthetist get their funding and I haven't considered it enough to have a perfect answer.” **(Participant 8)*** *“So I was going to say whether or not, is there a role for professional body, arguably yes. Is there a space for professional representative body like that? I'm not sure and there's a politician from 20 years ago called Michael McDowell, and he always said “it's one thing to say that there's a niche in the market, but you have to ask if there's a market in the niche. And if there's to be another body representing pharmacists, then that body will need to be funded and I don't know that pharmacists are ready to fund yet another organization. There already funding the PSI, the IIOP, the IPU.” (**Participant 4).*** *“I suppose they should represent everyone. I think fees will always be an issue for them. So then we'd have to look at the PSI are we still paying them the €400 a year and then do these guys want another €400 a year, so that won't work. So the PSI will have to come forward with you know, what do you do with our €400 because, I'd really like to know personally and you know because they are state funded….but I think that might be an issue, so it might be that the PSI fees would have to come down to help support this organization to balance out…..€400 is a lot every year to spend for something that just says MPSI, particularly like me who doesn't use it for any particular purpose……It's not that it allows me to practice, I can still do my job with or without it. So then if I need to move to somewhere else and maybe MPSI, I know that kind of moves with the new organization, because I don't need to be a member of a regulator, do you know what I mean. Why would I be happy to pay membership to a regulator, that makes no sense to me. The regulator should just regulate. So then the question is, well, why would I pay a fee to them, so I pay the fee to be a member of a society, but there's a piece. So there is a little bit of work to work that out.” **(Participant 9)***
	Membership & Inclusiveness	*“I think that access to a professional support body should be universal to all members of the profession. And that those members should not be compromised, you know, in terms of having to, you know, be a member of a union or be a member of something which could raise you know conscientious objection or and a moral dilemma.” **(Participant 13)*** *“[A] professional body across the profession. Now I think there may be some industrial pharmacists, who would complain and hospital pharmacist says nobody understands me, and community pharmacist feel nobody understands them, but if we started at that, wouldn't it be massive? You know, start small so don't try and boil the ocean, start with the concept and then you know if that ends up splitting into various different groups, but there's no reason why each area of practice couldn't lead their own professional agenda, because it would be owned by the profession, you would have nominations or an election process for the Community pharmacy professional agenda, the hospital pharmacy professional agenda, the industry or advanced practice or regulatory or academic, you'd have your committees and you would have the profession speaking from one voice. I think it would be massively destructive to start multiple professional bodies.” **(Participant 1)**.* *“So when you've got an entire profession, so singularly united on one common purpose, I think diluting their professional agenda by splitting it up is a dangerous thing to do, and I think a professional body going back to the point, with very good leadership, − would easily be able to support on all agendas, and in actual fact should support on all agendas. We need to get to a point that when anybody sees the medicine they think pharmacist, and ultimately whenever they see a medicine, whether it's been sold, manufactured, discovered, researched, clinical trial, delivered, whatever, dispensed, consumed. Whenever they see it, they think the professional body for pharmacy.” **(Participant 1)*** *“[Professional Body] that's for anyone who did a degree in pharmacy, basically**.”(Participant 9)*** *“If we're going to have a professional body, I would say they need to follow the principles of collective action and that would be to design and implement an initiative with the priority placed on equity, so you know sustainable improvements in community, not looking at being driven by a particular agenda be it as in education or regulation, you know it needs to be really equitable. So everybody that's involved from a pharmacy perspective all the stakeholders, the practitioners, need to feel that this is theirs. It needs to include community members in the collaborative so we would need pharmacists to be absolutely the ones that are most, you know, the people who are most directly and deeply affected by the problem. And patients would need to be involved in it too. And then we would need to co-create and, you know, work with cross sector partners, so that body would need to be able to collaborate with other medical practitioners with our DoH, with our HSE. It would need to use data to continuously learn, adapt and improve pharmacy practice. Understanding that you know collective impact isn't a solution, but it's rather a collaborative problem solving process, and we would need to cultivate leaders with unique system leadership skills.” **(Participant 1)*** *“So I think informing the body for inclusiveness purposes. I think you need to think of the patient facing and the non-patient facing roles and also develop those skill-sets like for example mentorship and leadership and coaching are coming so much more into practice that they're not separate to being a clinical pharmacist, there very much part of it.” **(Participant 11)*** *So I think to be inclusive, it's really, really important that all the different avenues are addressed as a professional body. It's a professional body for the profession, you know it's not just a hospital body or a clinical body, or so I think. I think when such a body would in theory be formed, I think it is really important that those aspects are considered in terms of say border or governance or oversight membership, exactly what it has to offer. **(Participant 11).*** *“Yeah I would just be afraid that you know something you know that hasn't got as many pharmacist might feel like, well, you know why would we bother, I mean what have we to get out of it? And so I don't know, I think the goal and mission would have to be very clear that it's all inclusive, and that one voice is just the same as any other voice then that we won't have. You know, you might have special interest groups where you know people, but that, if there isn't one voice, I think then what would happen is you wouldn't really get the buy in. And then government wouldn't really take it seriously anyway because they be like, well I mean your own profession doesn't really support you, so would we listen to what you have to say? So it would kind of be dead in the water, you know?” **(Participant 5)*** *“And you know, and obviously if there is a professional body for the profession as a whole, the vast majority of pharmacists practice in community practice, and you know therefore their needs might be very different to say somebody like me or somebody working say in specialist hospital practice. So I think I think the other thing is I think we're a small country with a small population of pharmacists, so we you know we need a system, that encompasses as many people as possible while still allowing for the diversion of roles so I'm not sure what would be the best solution to that.” **(Participant 6)*** *“Whereas really there are lots of opportunities in the kind of the merge points between the three sectors, where there is actually a massive amount of opportunity and I think the only way around it really is to get a body that represents absolutely anyone that is on the register as a pharmacist, so practicing in any capacity as a pharmacist, that would include academia as well, or those that diverge after into other roles. And so I think the special body should be able to offer something to anyone that has MPSI after their name and also the intern students come along the road behind us. Rather than just specifically looking after the people that fall into their neat little basket.” **(Participant 7)*** *“Just that I would see it as being a professional body for all pharmacists. Again, people have spoken to me about this, it became clear they think it's one subsection of our profession* versus *another and that's not it. A professional body is you know everybody on the register irrespective of how we practice or where we practice. And I'm 20, something years a pharmacist and I'm still ambitious for my profession. And I don't think we have as a profession, individuals have pushed the boundaries and individuals have grown our role and individuals have shown how important are skills, knowledge and experience arts the care of patients. But until we become a collective group that are pushing in that direction, we're not going to make ground quickly, or we get left behind, you know which, I would be so disappointed to see**.” (Participant 8)*** *But I don't see any problem with there being, pharmacists in a professional body, who don't actually work in a patient facing role. I mean as an academic, I don't directly contribute to patient care, but I like to think I could make some contribution to it, even if it's only through undergraduate education**. (Participant 10)*** *“And I think it would need to be, and I think you know we can look to the likes of PIER to see that there is a market for this and that there is, you know a cohort of people who aren't what you might call may be eligible or accommodated by what is there already. You know, so we would be very nice to see you know, a united professional body and you know, I guess I would like to see that with greater synergy across and within and between the members, you know. So that we could begin to see better synergies, for example, between pharmacists who might be in informatics, those who might be an industry, those who might be in regulation, those who are on the front line. You know that this body could actually lead to better cohesion across those sectors, and too better maybe you call it interdisciplinary and or intra-professional working, you know across that. Yeah it would be nice to see that all happening under one professional body.” **(Participant 13)*** *“Definitely spoken to one or two of them, they felt real pull, that they've had to come off the register and could no longer see themselves as MPSIs because of the CPD requirement. So I think if the MPSI bit moved somewhere else that would be really good for those people who still influence the industry, but may not be actively practicing within the industry. And I mean those elders as we'll call them, they have a lot to bring and they have a lot of wisdom that we shouldn't lose, and there's a lot of other pharmacists who equally because they're not in clinical practice, have come off the register, because the CPD requirements, because they don't see how it relates to them in their practice. And I think we've lost a lot of people who are pharmacists, who had stopped seeing themselves as pharmacists, because of that piece that came in when the CPD piece came in. So I think we have lost people and we could bring them back in if we had a representative body, where they could be part of that society and part of that culture and I think we would be nice to include them basically.” **(Participant 9)*** *“I think giving that to people and letting them use that is good for the profession, because it's good for the profession to have people doing jobs in loads of different sectors, which may not necessarily relate to their primary degree, but they draw from their primary degree to support them in their role in whatever capacity that job might be.” **(Participant 9)*** *And I think in the first instance you know, pharmacists should pay a certain amount for general membership, and then if they want to avail of additional services being credentialing, career structures, coaching, involvement, education, whatever, fellowship. Then they can pay incrementally for those specific tiers of service from the professional body.” **(Participant 1)*** *“I would not be obliged to be a member of the RPS in order to be a registered pharmacist. So I pay a fee to the GPhC and register, but it's voluntary as to whether I'm a member of the RPS…….Absolutely yeah it would certainly provide more money, but at the same time, I'm not sure that it should be a compulsion**.” (Participant 10).*** *“So in a sense should there be differing levels of fees? Maybe there should, but on the other hand up to it, it would depend very much upon what you are getting for the fee, and that will be what the Members themselves would then turn around and say well what am I getting for my money?” **(Participant 10)*** *“But I think if they're separate entities, you should pay a separate fee. Now I think if you just said we'll double the PSI fee, that will cause a lot of problems in the sense that I think you have to work out a structure. So if there's certain roles that this new body would potentially do the PSI currently do, or* vice versa *I think that whole structure would have to be delineated. Personally, just in theory, I'd be happy and I think it's important to pay the fee to both because they would have individual administrators, you know, and all the all the admin side that goes with any body…….I think in order to maintain their authenticity and autonomy, it's important that they're separate and then, as part of their charters, or their directorate or whatever document or if you like, is attached to each one in terms of how it functions.” **(Participant 11)*** *“I suppose that's a hard one to answer before you know what you're paying for. You know if you set up, it's a bit like a gym membership in some ways, if you're paying for, it would be a good or a bad example. But you know, if you're paying for certain things and you can see the difference in the value, I think it has to be value added. So if you're joining, maybe as a newly qualified pharmacist, you're going to have you know you're going to need a certain set of skills and competencies,* versus *somebody may be there 20 years and needs different input in the sense that if you're using more of the resources, then you could certainly look at it over time, the different types of membership, and I think it could be linked to need. So for example like in the Hospital Pharmacy Association for example, you can join as a student, as pre-registration pharmacist, as a fully qualified pharmacist, as a retired pharmacist and your needs and what resources you can get from the organization might be different. So I think you could certainly look at, not just have different fees for the sake of it, but if it's associated with different services in different resources as careers develop, or different areas, things like that, I think it's something that certainly could be scoped out. I don't know what the answer would be in terms of you know the absolutes, but I think there's scope there to look at to look at different aspects of what a body offers for different career stages.” **(Participant 11)*** *“I don't think mandatory supports membership and buy in. I think it does the opposite. I think if you have mandatory you might get a lot of names on the list and you might get a lot of disgruntlement, but you will not get engagement you know, whereas if something is optional and you've got a leader who will win over hearts and minds and who will make a compelling argument for the individual and collective benefits of being part of this club, you might think of it, I think you're much more likely to get better engagement. And then in terms of the fee and the fee structure yes, I, uh and again you know I'd need to have a look at their tiered levels, I'm not actually familiar with, you know what's within the different tiered levels of the of the RPS, but I guess I could imagine it being, you know, access to different resources.” **(Participant 13)*** *“I actually have no problem with a separate membership fee, I would have absolutely no problem with a separate membership fee, but I actually think the PSI membership fee needs to change. Because say for example, I pay exactly the same membership as my wife does, she's a practicing pharmacist, I'm not practicing pharmacist…….I am still a pharmacist, but I'm not in the patient facing roles. So I think that my registration status should be slightly different and the PSI don't maintain different registers, but I have no problem paying be it 200 euro or whatever to a professional body that would advocate on behalf of all pharmacists.” **(Participant 3)*** *“I would love if instead of the 380 that it was split up and you had to pay. I mean, nurses pay €100 a year…..we pay 380 and we don't even get a magazine…..I don't think I get anything except an email saying this has happened and that has happened….. but I'm not sure like really 380? I would prefer to say OK, well I'll give you 180 and I'll give the other 200 to an organization that runs I don't know CPD for me or that runs, you know, I don't know. And I don't how the IIOP is funded, by the way, I don't know that funding mechanism, so maybe it's funded through the PSI…….but I would happily, you know, think that, oh well, at least it's going to the betterment of the profession at least it's going to something like that where I can say, well, there's a tangible benefit. But I wouldn't at the same time leave it too optional, because I'd say if you do then again it'll be kind of like well, I'm always member of the IPU, so why would I need this, and so I think if you're serious about doing this then it probably does need to be funded through a kind of a mandatory mechanism, but I don't know how you would do that. Because if there was a whiff of the fees going down, then you know people would say well why or why are you taking this money the whole time anyway, so I'd like to see it being divvied between the two. Even though I want no crossover between the two, having said all of that. You know they have to be separate entities, but you know if there could be one funding mechanism, and then when it gets this point it just splits off and half goes here and half goes here, that would be great. I'd like to see that.” **(Participant 5)*** *“[I]f I wanted to attend like a webinar from I don't know consultant psychiatrist and might get. I think that could work……there could be different courses, like I think something that people have said to me is that they'd love a return to work as a pharmacist course or something like that. And maybe you know for an extra whatever you can get, these are as you say, maybe there's an added benefit. Like I, I think, as far as I know, IPU you know to join the Academy, there's a certain amount every year. But you have to pay, but uhm, I think it's like 150 euro but yeah, so that would be along the lines of what you mentioned**.”(Participant 5)*** *“So I would prefer it with voluntary because I think that people would have to decide if it was an organization that they were interested in joining and engaging with and there may be a part of you know people in particular roles in the profession where that may not be the case. So I would say voluntary.” **(Participant 6)*** *“Yeah, I think for anyone really it's going to be what you get back so, what are they going to offer, I remember years ago I use to be member of the RPSGB and then when I came home from the UK, I was able to keep a kind of on the register but not really practicing member. So they use to post out the pharmaceutical journal every month to me, and like you, if I decided then that I wanted to go back on the register, it was a much quicker path then kind of coming back in from the cold we'll say. So yeah I think kind of a few bits and pieces like that that could be quite useful, especially for those that are maybe going into some industry roles, where you know if they've developed along a particular pathway and they're not really doing pharmacist work anymore, but still be beneficial for them to stay on the periphery of registration. So I think, yeah yes maybe three options would be useful.” **(Participant 7)*** *“Yeah, I mean, so if we're saying that there is a lump sum I pay every year and then that's split between, that some of that funding goes to the regulator and then or some of the funding or all the funding goes to the College or the professional body, and then some of it is split out and provided to the regulator to do their work. I suppose it would depends on what is the value added. Tiered would work if you have a real value proposition. It's really difficult to see in the Irish context how we would be big enough to sustain that model. We are very small and there's only you know we're not….we don't even have 10,000 members.” **(Participant 8)** “That's a bit further down the line, I would have thought, at the moment until what that professional body does, I don't really know much about tiered system, I think to have to be to be a representation body for pharmacist there is a fee and that is. I mean I wouldn't see that there's tiered, I don't see, that's years down the line to see how it is. I mean I, and that's my thoughts. I mean we don't have a structure as I know it's, and it's also unfortunate in the Irish pharmacy sector that the last 50 years we, the profession pharmacist, don't pay. They don't. They pay one fee and they just pay it and they never question is. Whereas I think in other professions in Ireland like medicine and dentistry and so on, they recognize the need to pay fees, to do CPDs, and so it's a very, it's a different mindset and we've lost that kind of recognition in, and for a generation now, where the assumption is that you're paying. Well, we hadn't put a value on it before and it would be very hard to change that mechanism. Uh, so and there's a sense that that's pharmacists are expecting to guess what they need out of it. Now I would assume as an example maybe the mechanism that you could do it is that if you want to be regulated, and if you want to be a supervising pharmacist, you need to have a college of pharmacy accreditation so you need to be an …… So it might become voluntary in that case that you, in order to be able to do this, you need to do your CPD points, you need to do this. So you might be charged like there's different business model. My feeling is I wouldn't go near tiered, I would just understand, but in my experience how that might work is that you would pay a set fee to be a member and again my feeling is either College of Pharmacy or as I see it, the PSI should, the title should transition over, and you pay for that and that allows you access to the IIOP and that allows you to record your CPD and then that's a mechanism that if you do want to be regulated and to be licensed and so on through the regulator as an independent pharmacist, regulated like in the UK that that that you would have to pay. You need both basically.” **(Participant 2)*** *“The honest answer is I don't have enough understanding currently to know how it works in other professions. You know, like to me, the PSI, for me I don't understand the workings of the PSI from a financial perspective, I don't know where they get their money from. I presume the state pays for them, and pays the salaries of the people who work there. I presume I actually have no idea. But you know my MPSI is to pay for me to be a member of a pharmaceutical society of Ireland, but the pharmaceuticals society of Ireland, is currently a regulator so you know, I'm paying to be regulated, that's kind of madness on one level. But I think there would have to be in equilibrium there between the two, because pharmacists are awful tight, and they would be like, why would I be paying for that crowd and for that crowd? That makes no sense to me, so I think there has to be a balance struck there between the two, because you won't get buy in, and then you won't get membership…….I suppose that's a detail that's way, way down the line. At this point is probably more that the ethos and you know what conceptually would it work and how it would work and why would it work. And I think you know being separate from the regulator is important.” **(Participant 9)*** *“I'd be interested to see how much of your €380 that you gives to PSI goes to the IIOP and I think say you could…say for example, this is just a guesstimate now that if it was 25% of that fee goes across the IIOP, I think the cleanest thing to do would be decreased the PSI fee by 25% and that we would pay separate fee then into the IIOP directly or the 25% and that there would be a separate revenue streams coming into them and have that investment.” **(Participant 7)*** *I mean the fees have to be separate I can't see that the PSI that the fee if the remit and organizations governance structure is stand alone as it is. And in fact I would argue is negligent of the PSI to be taking fees when they're serving no purpose of me as an example. I'm not regulated, well I'm accredited program, but it's not a requirement of my job to do that. So taking the fees and that grounds. Now, if I was practicing in pharmacy and selling products and so on and acting in accordance with the Pharmacy Act certainly then I should be voluntarily done. So I can't see any situation where the PSI fee could be split, it just doesn't make sense to me, maybe in the short term. And I can't see from a corporate or legal perspective, where the lawyers in the PSI, the regulator as we call at the moment would agree to such a scenario. I would argue are they equally happy that they're taking funding where they're not regulating in many cases. So I guess and they shouldn't be taking fees, as I see it as a regulator for people who just want to be called MPSI, a member of a professional body. They should only be charging fees for those who need to be regulated, so this is exactly that's as I see it, there funding model has to match that and so to answer your question, I think they have to be stand alone and the in both cases there voluntary as I see, if you don't want to practice pharmacy, you don't have to pay regulator, but it's a legal requirement that if you are to practice in a regulated unit, be their regulated pharmacy, which is essentially an inspected facility, then you need to be and if not you can be regulated. Whether you want to be member of professional is purely voluntary and it needs to be standalone.” **(Participant 2)*** *And the RPS has faced some degree of difficulty, because whilst it had some resources, the tasks that it is taken on are really quite diverse and quite expensive to perform, and they have, you know. **(Participant 10).*** *But we have not got that right, but the problem going back to fees is, pharmacist are not willing to pay fees and in UK RPS does have a problem with membership and income, and even though they're doing very good work they have a lot of critics internally within the UK. Lot of people see them as too elitist and too focused on, you know, GP practice and hospital to the exclusion of the majority pharmacist who are in community pharmacy**.(Participant 12)*** *“I'm very familiar with the RPS and with the work that it does, as I say it does high quality work, but its membership is not growing. If anything it's going backwards, it has struggled financially for years, even though it's like an incredibly wealthy organization, but it's run at a deficit for years because pharmacists aren't universally excited about the idea of funding it, and huge number of the pharmacists that I know in the UK feel that it is divorced from their interests.” **(Participant 4)*** *“I think it would take a lot of energy and it would have to be funded. And who's going to pay for it. I mean, certainly I don't know where that funding would come from. And if you say Oh well, maybe it'll come from the regulator, because that's what they did in the UK as far as I remember anyway, they kind of split it, but you know you had to sign up to both. But I think initially the Royal Pharmaceutical Society really struggled because people were like, well, what's the added value here like. I mean, I have to pay my registration fee, so that's a no brainer, but like why do I have to sign up to the RPS?” **(Participant 5)***

## Appendix A Interview topic guide

1. What does professional representation mean to you in general?
2. How do you feel about the suggested creation of a Professional Body of Pharmacy?
3. Do you feel that pharmacists in Ireland are currently sufficiently represented by existing organization?
4. Do you feel that it is important for Pharmacy to be represented independently of regulation?
5. If a Professional Body of Pharmacy was created, what do you feel its key roles and responsibilities should be?
6. Do you feel that a Professional Body of Pharmacy should be responsible for accreditation of educational programmes or should that role remain within the PSI?
7. What model do you think a Professional Body of Pharmacy, if formed, should take? **Prompt:** Incorporating existing organizations or alongside some/all, similar to the UK model (describe if participant unaware).
8. What type of leadership qualities would you consider necessary for a proposed bodies?
9. What are your views of membership fees for a Professional Body of Pharmacy?
  a. Should membership be included in PSI registration fess or a separate fee?
  b. If a separate fee, do you feel that it should be a single fee for all or a tiered membership model?
10. Do you feel that membership of a Professional Body of Pharmacy alone would sufficient for non-patient facing pharmacists?
11. Is there anything else that we have not yet talked about that you think would be useful or good to discuss?

## Appendix B COREQ (Consolidated Criteria For Reporting Qualitative Studies) statement: 32 item checklist*

**Domain 1: research team and reflexivity:**

**Domain 2: study design:**

**Domain 3: analysis & findings**
